# Implementing digital devices to increase mobility training for people receiving inpatient rehabilitation: protocol for a feasibility hybrid type II randomized controlled trial

**DOI:** 10.1186/s40814-023-01298-y

**Published:** 2023-04-25

**Authors:** Nisha Aravind, Daniel Treacy, Sakina Chagpar, Lisa A. Harvey, Joanne V. Glinsky, Catherine Sherrington, Leanne M. Hassett

**Affiliations:** 1 Sydney School of Health Sciences, Faculty of Medicine and Health, University of Sydney, Sydney, Australia; 2 Physiotherapy Department, Prince of Wales Hospital, South Eastern Sydney Local Health District, Sydney, Australia; 3 Institute for Musculoskeletal Health, The University of Sydney/Sydney Local Health District, Sydney, Australia; 4 Sydney School of Public Health, Faculty of Medicine and Health, The University of Sydney, Sydney, Australia; 5 John Walsh Centre for Rehabilitation Research, Faculty of Medicine and Health, The University of Sydney Northern Clinical School, Sydney, Australia; 6 Department of Health Sciences, Macquarie University, Sydney, Australia; 7 Sydney Musculoskeletal Health, The University of Sydney, Sydney, Australia

**Keywords:** Implementation, Rehabilitation, Technology, Feasibility, Mobility, Physiotherapy

## Abstract

**Background:**

People with mobility limitations can benefit from rehabilitation programs incorporating intensive, repetitive, and task-specific exercises using digital devices such as virtual reality gaming systems, tablet and smartphone applications, and wearable devices. The Activity and MObility UsiNg Technology (AMOUNT) rehabilitation trial (*n* = 300) showed improvements in mobility in people using these types of digital devices in addition to their usual rehabilitation care when the intervention was provided by an additional study-funded physiotherapist. However, it is not clear if this intervention can be implemented by hospital physiotherapists with a usual clinical load. The AMOUNT Implementation trial aims to explore the feasibility of conducting a large-scale implementation trial.

**Methods:**

A pragmatic, assessor blinded, feasibility hybrid type II randomized controlled trial will be undertaken at a public hospital in Australia. There will be two phases. Phase I (Implementation phase) will involve implementing the digital devices into physiotherapy practice. Physiotherapists from the rehabilitation ward will receive a multifaceted implementation strategy guided by the Capabilities, Opportunities, Motivation-Behaviour (COM-B) theoretical model. The implementation strategy includes identifying and training a clinical champion; providing digital devices and education and training; facilitating use of the devices through clinical reasoning sessions and journal clubs; and audit and feedback of exercise dosage documentation. Phase II (Trial phase) will involve randomising 30 eligible inpatients from the same ward into either usual care or usual care plus an additional 30 min or more of exercises using digital devices. This intervention will be provided by the physiotherapists who took part in the implementation phase. We will collect data on feasibility, implementation, and patient-level clinical outcomes. The three primary outcome measures are the extent to which physiotherapists document the dosage of exercises provided to participants (feasibility criteria: exercise practice sheets complete for ≥85% of all participants); ability to recruit participants; and fidelity to the protocol of using digital devices to prescribe exercises (feasibility criteria: average of ≥ 30mins per day for > 50% intervention participants).

**Discussion:**

This feasibility study will provide important information to guide the planning and conduct of a future large-scale implementation trial.

**Trial registration:**

Australian and New Zealand Clinical Trial Registry; ACTRN12621000938808; registered 19/07/2021. Trial sponsor: Prince of Wales Hospital. 320–346 Barker Street, Randwick, NSW, 2031, Australia. Protocol version: 6.2 7th April 2021.

**Supplementary Information:**

The online version contains supplementary material available at 10.1186/s40814-023-01298-y.

## Introduction

Rehabilitation is a global priority due to the aging population and the increasing number of people living with the consequences of disease and injury [1]. The importance of rehabilitation has been highlighted in the wake of the long-term effects of COVID-19 [2]. It is well accepted that large numbers of repetitive task-specific exercises should be part of most rehabilitation programs which aim to improve function, particularly rehabilitation programs for the aged and those with neurological conditions [3–5]. Repetitive task-specific exercise refers to “an active motor sequence performed repetitively within a single training session, with practice aiming towards a clear functional goal”(5). For example, gait training - where the function of walking is trained by a high number of repetitions within a session. However, observational studies in high-income countries have found that people undertaking inpatient rehabilitation do not perform large numbers of repetitive task-specific exercises [6–8]. Thus, strategies need to be investigated to increase the amount of repetitive practice patients perform within inpatient rehabilitation wards. One strategy may include the use of affordable digital devices to provide exercises and monitor the amount of practice and provide feedback. For example, virtual reality gaming systems such as *Xbox Kinect* (Microsoft, Washington, USA) provide an inexpensive way of encouraging standing and stepping practice, and wearable devices such as the *StepWatch* (Modus Health, LLC) can be used to monitor physical activity and provide feedback on the number of steps taken a day. Digital devices can be used as part of one-on-one therapy sessions, or to encourage patients to exercise in a semi-supervised or group environment, or on their own.

We undertook a clinical trial (*n* = 300) using digital devices to provide exercises in rehabilitation. This trial was called The Activity and MObility UsiNg Technology (AMOUNT) rehabilitation trial. We demonstrated clinically important improvements in mobility from baseline to 3 weeks and 6 months in people using digital devices for mobility exercises and overall physical activity in addition to their usual rehabilitation care [9]. Mobility exercises included standing up from a chair, stepping in different directions, and weight shift in standing while playing games using virtual reality or following a digital exercise program.The digital devices were prescribed by a physiotherapist and the prescription was tailored based on patients’ mobility limitations and preferences. The core element of the intervention was 30–60 min of additional task-specific mobility activities 5 days per week using a range of digital devices to enable tailoring and progression. The digital devices included virtual reality gaming systems, tablet and smartphone applications, and wearable devices. Importantly, the participants enjoyed using the digital devices and most rated the devices highly in measures of usability [9].

Although the first trial, the AMOUNT rehabilitation trial, was a pragmatic trial undertaken in “real-world” clinical settings, the exercises with digital devices provided as part of the intervention were delivered by physiotherapists employed using study funds. To take implementation of this approach to the next level, we need to determine if physiotherapists employed by hospitals can provide exercises using digital devices in addition to their usual therapy and whether devices used in this way improve mobility outcomes. Therefore, we will conduct a feasibility hybrid type II randomized controlled trial [10, 11]. This trial design will enable us to test trial processes to guide the design of a future large-scale implementation trial, pilot strategies to encourage and support physiotherapists to use digital devices as part of their therapy sessions and explore effects of our approach on implementation and patient outcomes. The exercises prescribed with the digital devices will be performed either under the direct supervision of a physiotherapist, as part of a class or in a semi-supervised environment. The digital devices will also provide a way of encouraging patients to practice on their own if deemed safe to do so by their physiotherapist.

The primary aim of this study is to determine the feasibility of conducting a large-scale implementation trial. Specifically, we will determine the feasibility of the following:

1. Collecting dosage data on the amount of repetitive task-specific practice patients receive as part of therapy;
2. Recruiting participants in the trial;
3. Fidelity to the protocol for using digital devices to prescribe appropriate exercises. Specifically, whether physiotherapists can provide 30–60 min of extra therapy on top of usual care using an array of digital devices appropriate for enhancing mobility.

The secondary aims are to:

1. Determine the processes, resource use and management systems required to enable the conduct of a large-scale implementation trial
2. Explore impacts on patient-level outcomes to guide the future trial
3. Explore implementation outcomes and determinants on implementation success in terms of physiotherapists’ capability, opportunity, and motivation to implement digital devices in rehabilitation.

## Methods

### Design

**Fig. 1:**
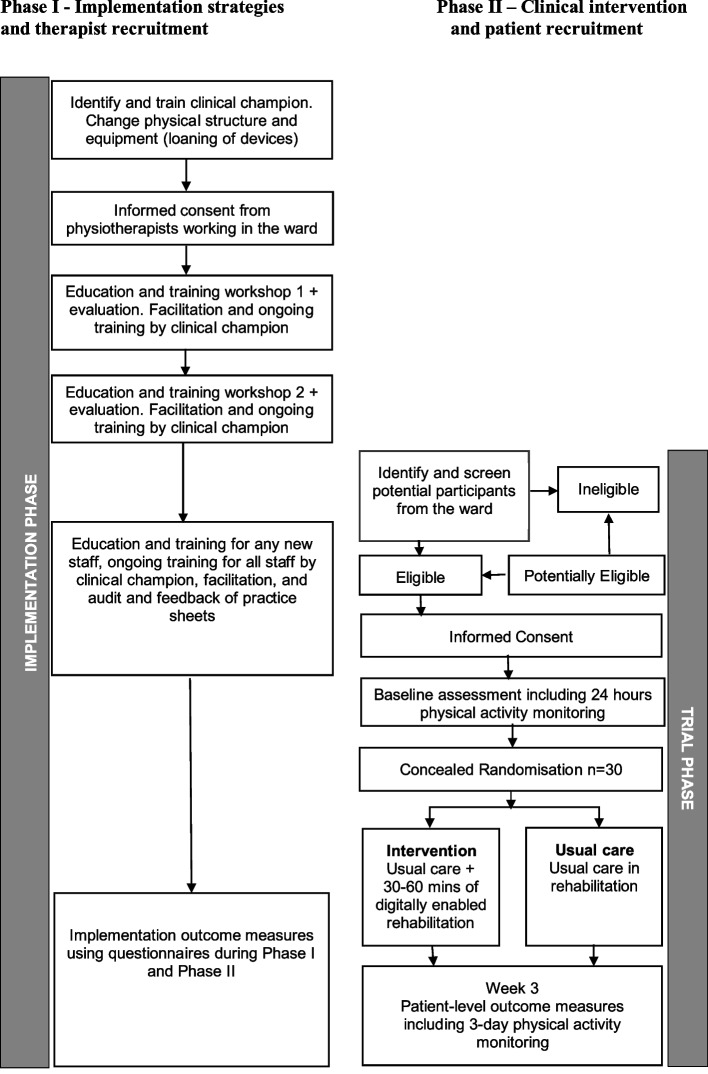
Flow diagram for the trial

A feasibility hybrid type II randomized controlled trial will be undertaken. There will be two phases: phase I (the implementation phase) and phase II (the trial phase). Phase I will involve delivering strategies to encourage and support the physiotherapists of a general rehabilitation ward to use digital devices as part of their physiotherapy sessions. Phase II will involve randomising patients of the same ward to the intervention or control group. The design of the trial is presented in Fig. 1. The trial will be reported according to the Consolidated Standards of Reporting Trials (CONSORT) statement for randomized pilot and feasibility trials [12], the Standard Protocol Items: Recommendations for Interventional Trials (SPIRIT) statement [13] (see Additional file 1) as well as the Template for Intervention Description and Replication (TIDieR) checklist for intervention description [14]. The trial has been prospectively registered in the Australia and New Zealand Clinical trial registry, ACTRN12621000938808 and approved by South Eastern Sydney Local Health District Human Research Ethics Committee, 2019/ETH13444.

### Setting, therapist-participants, and patient-participants

#### Setting

This study will be conducted in the general rehabilitation ward of Prince of Wales hospital in Sydney, Australia. The ward is a 14-bed adult inpatient ward. Eighty percent of the patients have a neurological condition (mostly stroke), and 20% have other non-neurological conditions such as deconditioning, amputations, and post-cardiac or orthopaedic surgeries. The ward provides a comprehensive multidisciplinary rehabilitation program to patients admitted to the ward through a team comprising of rehabilitation and neurological consultants, nursing staff, physiotherapists, physiotherapy assistants, occupational therapists, occupational therapy assistants, speech therapists, clinical psychologists, neuropsychologists, social workers, and dieticians. The hospital is a teaching site for many universities and the rehabilitation ward usually has students from different disciplines undertaking clinical placements.

Physiotherapy staff working in this setting include full-time, part-time, and job-share roles and includes a combination of senior and junior rotating physiotherapists, allied health assistants and physiotherapy students. The physiotherapy program is provided from Monday to Friday 9am to 4 pm in the physiotherapy gym. There is also a limited Saturday service from 9am to 4 pm provided by a physiotherapist and an assistant. Usually, each patient is timetabled to receive an hour of one-to-one supervised therapy in the morning and in the afternoon. The physiotherapy gym follows an open-door policy where patients can stay back in the gymnasium after a scheduled therapy session to do more physiotherapy in a semi-supervised environment as needed. There are also group sessions where patients exercise together. All patients are encouraged to do as much practice as possible by themselves outside formal therapy sessions with the support of family and friends if necessary.

### Participants

There are two participant groups, namely, the therapist-participants who will be supported and encouraged to use the digital devices with their patients through a range of implementation strategies, and the patient-participants who will use the digital devices as part of the therapy provided to them by the physiotherapists. The therapist-participants will be included in Phases I and II, and the patient-participants will be included in phase II. It is not possible to blind either participant group due to the nature of the intervention and implementation strategies.

Therapists will be eligible to participate in phases I and II if they.

- 1.Are physiotherapists who work at the Prince of Wales hospital
- 2. Are employed full-time or part-time and are permanent or casual, or rotate through the rehabilitation ward during the study period.

University physiotherapy students will not be included as participants in either phase I or II, but they may assist in the delivery of the clinical intervention in phase II under the supervision of physiotherapists enrolled in the study. The students will also be provided with education and training in the use of devices within their first week of placements.

Patients will be eligible to participate in phase II if they

- 1. Are admitted to the general rehabilitation ward of Prince of Wales hospital and are at least 18 years old
- 2. Have reduced mobility (Short Physical Performance Battery (SPPB) score of less than 12)
- 3. Are deemed by a clinician to have the potential to improve their mobility
- 4. Have a likely life expectancy of more than 6 months
- 5. Have an anticipated length of stay of ≥ 21 days after the date of randomisation

Patients will be ineligible to participate in phase II if they

- 1. Have cognitive impairment (Mini Mental State Examination (MMSE) [15] < 24 or Montreal Cognitive Assessment (MoCA) [16] < 18) unless their caregiver is present, and willing and able to use digital devices with the patient
- 2. Have insufficient English language skills to participate in the intervention without an interpreter
- 3. Have inadequate vision to use the digital devices
- 4. Have a medical condition(s) precluding exercise (e.g., unstable cardiac disease, uncontrolled hypertension, uncontrolled metabolic diseases, large abdominal aortic aneurysm, or a weight-bearing restriction).
- 5. Inability to balance safely in standing despite the assistance of a staff member or the nominated support person or walking aid.

### Recruitment and allocation

#### Therapist-participants for phases I and II

A member of the research team will provide physiotherapists of the rehabilitation ward with verbal information about the two phases of the study and provide them with a participant information sheet and consent form. Written informed consent will be obtained.

#### Patient-participants for phase II

All patients admitted to the rehabilitation ward will be screened against the eligibility criteria by the site coordinator, who will seek advice on patient eligibility from the multidisciplinary rehabilitation team as needed. Details will be recorded into a password protected screening log in Excel. Potential participants who may not be eligible at the first screening will continue to be screened during their inpatient stay and will be invited to participate in the study if subsequently found eligible. Patients who are eligible will be provided verbal information about the study. If the patient is interested in taking part in phase II of the trial, they will be provided with a written participant information sheet and consent form to read. All participants will provide informed written consent before recruitment into the study. Informed consent will be obtained by a researcher who is not responsible for the clinical care of the patient.

Patient-participants will be randomized to an intervention or control group after completion of baseline measures (including 24-h physical activity monitoring). Each participant will be assigned a study ID number and all future documents relating to a participant will use this study number. A variable block randomization schedule will be prepared using the Sampsi command in STATA v14 by a researcher not involved in patient recruitment. The randomization schedule will incorporate stratification for baseline mobility (SPPB score < 6 vs. ≥ 6) and health condition (neurological vs. non-neurological). The trial will use a centralized web-based randomization system stored on REDCap (Research Electronic Data Capture). This will ensure concealment of allocation to groups and an auditable process.

#### Details of the interventions provided to both patient groups during phase II

### Usual care

Patient-participants in both the intervention and control groups of phase II will receive usual inpatient rehabilitation care. All participants will be assessed by a physiotherapist who will prescribe a series of task-specific exercises to be performed as many times as possible according to their impairments and activity limitations (e.g., practice of standing up or stepping). The conduct of these exercises will be supervised by a physiotherapist, a physiotherapy assistant or physiotherapy student under the overall supervision of a physiotherapist, or by a family member who is educated by a physiotherapist to do so. As is standard practice, the exercises will be individually tailored and progressed. Participants will also receive feedback about their performance, and the exercises will focus on functionally relevant goals. The feedback will be provided in the form of knowledge of performance (e.g., centre of mass movement while shifting weight in standing during game play) and knowledge of results (e.g., game score) Participants will also receive assessment and management by other members of the multidisciplinary team as required. The exercises provided during usual care will be documented by the physiotherapist or physiotherapy assistant/student in an exercise practice sheet. The exercise practice sheet will have space to document details about the type of each exercise, number of repetitions of each exercise, and the active time spent on each exercise. If participants can exercise independently, they will be encouraged to also document this in their exercise practice sheets.

### Rehabilitation using digital devices (intervention)

Patient-participants randomized to the intervention group of phase II will receive individualized therapy utilising digital devices to enhance mobility and overall physical activity in addition to their usual care. The selection of digital devices are similar to that used in the AMOUNT trial [9] and will be the same as intended for the large-scale implementation trial in the future. Digital devices will be selected according to an intervention protocol that matches different exercises on different devices to common mobility limitations, as well as the physiotherapist considering participant impairments (e.g., upper limb weakness), goals and device preferences (See Additional file 2). The digital devices include rehabilitation and recreation virtual reality gaming systems, tablet and smartphone applications, and wearable devices. Each device provides feedback about task performance, enables individualized tailoring and progression of exercise or physical activity, promotes progress towards a functionally relevant goal, and is relatively inexpensive (see Additional file 3).

Physiotherapists responsible for the day-to-day therapy will teach the participants to use the digital devices and develop individualized exercise programs that enable participants to use the digital devices during and/or outside formal therapy sessions according to the participant’s capability, motivation, and goals. The exercises may or may not require the direct assistance of a physiotherapist and/or family members/friends, nursing staff, physiotherapy students or hospital volunteers. The physiotherapists will aim to ensure participants receive an additional 30–60 min of exercise with the digital devices, five or more days per week for 3 weeks. Our previous work has found this amount of additional practice using digital devices to be feasible and enjoyable for patients [9, 17]. Table 1 provides further detail of the intervention using the TIDieR checklist.

**Table 1:** TIDieR Checklist describing the clinical intervention

Checklist item	Intervention group	Control group
Brief name	Digitally enabled rehabilitation in addition to usual care	Usual care
Why	AMOUNT study [9] showed improvements in mobility with digitally enabled rehabilitation in addition to usual care when the intervention was provided by a research physiotherapist external to the service. This study will test whether the clinical intervention can be delivered by physiotherapists employed within a service in addition to the usual rehabilitation	Pragmatic trial design
What	Procedures: Patient-participants will be prescribed exercises targeting mobility using digital devices in addition to usual physiotherapy exercises Materials: The digital devices include: Recreational devices like: Nintendo Wii (Nintendo, Kyoto, Japan); Xbox Kinect (Microsoft, Redmond, Washington, USA); and Rehabilitation devices like: StepWatch (Modus Health, USA); LusioMate (Lusio Rehab, Sydney, Australia); Clock Yourself (iphone application, Brisbane, Australia); PTX app (iphone application, Sydney, Australia); Humac Balance System (CSMi Solutions, Stoughton, Massachusetts, USA); Fysiogaming (Doctor Kinetic, Amsterdam, The Netherlands)	Procedures: Patient-participants will receive usual inpatient physiotherapy care including assessment and prescription of tailored task specific and strengthening repetitive exercises (e.g., practice of standing up, stepping and gait training), usual care also includes assessment and tailored management by medical specialists, nurses, occupational therapists, speech pathologists, social workers, and dietician Materials: All the equipment used in a regular physiotherapy gym including treadmill, bike, weights and theraband
Who provided	Physiotherapists employed in the rehabilitation ward	
How	One-on-one, semi-supervised, independent and group sessions according to ability and safety	
Where	Inpatient physiotherapy gym and in their rooms on the rehabilitation ward	
When and how much	30 to 60 min additional therapy using digital devices daily for five or more days per week for 3 weeks	Monday to Friday, typically 2 × 1 h sessions/day, for 3 weeks. A Saturday session may be provided if assessed as a clinical priority
Tailoring	Tailored for each participant by physiotherapist according to their mobility limitations, goals, and preference of devices	Tailored for each participant by physiotherapist according to their impairments, activity limitations and goals
Modifications	Modifications to the clinical intervention will be documented throughout the trial	None
How well	Fidelity measures will be collected as part of the implementation evaluation	

The exercises provided using digital devices to the intervention participants will be documented in a second set of exercise practice sheets designed specifically to capture the trial intervention. The exercises will be documented by the physiotherapist or physiotherapy assistant/student. Like the exercise practice sheets for usual care, the exercise practice sheets for the intervention will also have space to document the type and number of repetitions of each exercise and the active time spent on each exercise. If participants are capable of doing exercises independently, they will be encouraged to document them in their exercise practice sheets.

### Multi-faceted implementation strategy

All recruited physiotherapists during phase I of the study will receive implementation strategies to support them in providing additional exercises using digital devices into clinical practice. The multi-faceted implementation strategy has been developed using the Capability, Opportunity, Motivation–Behaviour (COM-B) model of behaviour change. This model categorizes barriers and facilitators to implementation. The implementation strategy has also been developed using the Behaviour Change Wheel to identify intervention functions of education, training, enablement, and modeling to address identified barriers [18]. Prior qualitative work done as part of the initial AMOUNT rehabilitation trial identified likely influences on implementation [17, 19]. The implementation strategies have been defined based on the Expert Recommendations for Implementing Change (ERIC) taxonomy [20] and include (i) identifying and training a physiotherapist on the ward to be a clinical champion, (ii) changing physical structures and equipment (loaning of devices), (iii) providing education through workshops (face-to-face or online), (iv) conducting ongoing training in device use, (v) conducting facilitation (resource provision, clinical reasoning sessions and journal clubs), and (vi) conducting audits and providing feedback of dosage data (using the practice sheets). Table 2 provides further detail of each implementation strategy including the proposed mechanism of change using the COM-B model. A training log will be kept by the clinical champion to monitor delivery of the implementation strategies.

**Table 2:** Description of multi-faceted implementation strategy delivered to teams of physiotherapists mapped to behaviour change wheel [27]

Implementation strategy [18]	Mode of delivery/where/length/who delivered	Time frame	Proposed mechanism of action			Intervention content
			Barriers targeted	COM-B domains [27]	COM-B intervention functions	
Identify and train clinical champion	Face-to-face/onsite and at University/fortnightly 3 months/LMH, DT, and SC	Prior to implementation phase	Skill in how to use devices; solve technical issues; clinical reasoning for device choice; access to expert in device use through working day	Physical capability; automatic and reflective motivation	Education; training; enablement; and modeling	Identify and train a clinical champion who is a physiotherapist at the rehabilitation ward (NA). NA trained in use of devices prior to commencing implementation phase with clinicians
Change physical structure and equipment	Face-to-face/on-site/length of study/LMH, DT, SC, NA	Prior to implementation phase	Access to a range of devices; technical problems impact patient and therapist motivation; time taken to turn on and set up devices	Physical and social opportunity	Restructure the environment	Loaning of required equipment to the hospital. Set up devices in collaboration with hospital IT department in rehabilitation gym. Develop processes for setting up equipment each day
Education	Face-to-face and remote/onsite and via zoom/1–2 h/LMH	Two workshops at beginning phase I 3 months apart and throughout study	Understanding of potential benefits of device use to patient and therapist; knowledge of current evidence of effectiveness	Psychological capability; reflective motivation	Education; Persuasion	Provision of education on current evidence of using digital devices in physiotherapy including results of AMOUNT rehabilitation trial; case studies from the AMOUNT trial
Conduct ongoing training (make training dynamic)	Face-to-face/onsite/as needed/NA, SC, LMH	First workshop and throughout study	Access to hands-on training and resources to support use; skill in how to use different devices and select games/exercises; optimize use for outcomes and ensure safety	Physical and psychological capability; automatic and reflective motivation; physical opportunity	Training; persuasion; enablement	Hands-on practical session using different devices led by clinical champion. Scheduled and ad hoc sessions
Facilitation	Face-to-face/onsite/45–90 min sessions/NA, LMH, DT	Throughout study	Access to training and resources to support use; clinical reasoning for device choice	Psychological capability; reflective motivation; physical and social opportunity	Education; enablement; modeling; persuasion	Clinical reasoning and journal club sessions in a team setting, access to resources (AMOUNT intervention protocol, cheat sheets device use)
Audit and provide feedback	Face-to-face/onsite/1 week recording/NA, LMH, SC	Twice during implementation phase	Habit of recording dosage of practice; lack of understanding use of dosage data for clinical and research purposes; not routinely done by team	Psychological capability; social opportunity; reflective and automatic motivation	Education; modeling; persuasion	Audit recording of dosage on exercise practice sheets for a 1-week period. Provide individual feedback to therapists and summary feedback to the team by clinical champion

Key: *IT-* Information Technology

### Outcome measures

There are a range of outcomes measures that will be collected during phases I and II. They are classified as either.

1. Feasibility outcome measures of trial processes, resource use, and management systems (see Table 3), or
2. Implementation outcome measures and determinants of implementation (see Table 3), or
3. Patient-level clinical trial outcomes (see Table 4).

The feasibility outcome measures capture different aspects of the processes, resource use, and management required to run a large-scale implementation trial. For example, data on the processes include screen failures and reason for potential participants declining to be involved in phase II of the trial. Data on resource use include the number of times the digital devices did not work or needed to be repaired. They also include data on the amount of training the physiotherapist required. Data on management includes the number of digital devices from which dosage data were successfully retrieved, and who collected what data.

The feasibility outcome measures also capture patient- and implementation measures of feasibility during both phase I and phase II. These include measures such as patient safety, treatment fidelity, and the success of the implementation strategies in improving physiotherapists’ capabilities, opportunities, and motivation to use the digital devices.

Where appropriate, feasibility criteria have been specified for each outcome measure to guide any modifications required to the study design, implementation strategies and intervention for the proposed large-scale implementation trial (see Table 3).

**Table 3:** Feasibility outcome measures

Feasibility outcome measures	Details	Feasibility criteria
1. Processes		
a. Recruitment rate	Number of participants recruited into the study per week (a **PRIMARY** outcome)	Nil
b. Eligibility criteria	Proportion of patients screened who were eligible and ineligible, and reasons why patients were ineligible	Nil
b. Refusal rate	Proportion of people screened who were eligible who declined taking part in the study and the reasons why they declined	Nil
d. Retention rate	Number of participants who have a 3-week assessment (primary outcome timepoint) completed prior to discharge between 2 and 4 weeks post-randomization)	≥ 85% completed for all outcome measures except activity monitor (≥ 80%)
2. Resource use		
a. Equipment	•Number and cost of devices + consumables/support equipment (e.g., data cards) •Number lost/broken devices needing replacement	Nil
b. IT	How many times devices did not work, time needed to fix, how and who it was fixed by and cost of fixing it	≥ 75% devices work for at least 75% of trial time
c. Staff	•Total FTE staff within the physiotherapy rehabilitation team that are participating in the trial •Number of physiotherapists needed training over course of project and number of hours spent training them in using digital devices •Number of physiotherapy students who required training (total blocks and total students) over course of project and number of hours spent training them in using digital devices •Number of training sessions missed by staff and students and reasons why •Number of staff trained and conducting screening, recruitment and outcome assessments	Nil
d. Training physiotherapists to embed digital devices	•Time and hours spent by clinical champion and study investigators in staff training •Number of workshops/journal clubs/clinical reasoning/audit and feedback activities performed	Nil
3.Management		
a. Dosage	•Percentage of exercise practice sheets reflecting usual care and digital device that are complete and legible with repetitions recorded (a **PRIMARY **outcome) •Number of devices that dosage data were successfully retrieved from •Who collected and how were dosage data collected for technology use?	•Exercise practice sheets complete for ≥ 85% of all participants
4. Patient- and implementation outcome measures		
a. Safety	•Number and type of minor and serious adverse events (e.g., falls) and circumstances of event when using digital devices during supervised/semi-supervised/out of therapy practice collected from hospital incidence reporting system	Nil
b. Estimation of effectiveness	•Between group difference and 95% CI for each patient-level outcome measure at 3 weeks post-randomization (these are outlined in Table 4)	Nil
c. Success of implementation	•Fidelity: average total active time in minutes per day using digital devices for mobility tasks (a **PRIMARY** outcome) •Number of days over 3-week intervention technology not used by participants and reasons why (e.g., patient refusal, technology not working, staff shortages) •Device use: percentage of participants each device was used with; average number of different devices used per participant; percentage of devices used per participant where it was progressed (e.g., game level, repetitions, sets, time) •Type of practice: percentage of participants each mobility limitation was trained with devices; average number of different mobility limitations trained per participant •Percentage of staff trained that participated in prescribing and delivering rehabilitation using digital devices	Average of ≥ 30 min per day for > 50% intervention participants; 0 min per day for > 90% control participants (to confirm no contamination)
d. Determinants of implementation success	•Change in physiotherapists’ capability, opportunity and motivation to prescribing and delivering rehabilitation using digital devices, measured using study specific questionnaire (see Additional file 4), developed based upon the COM-B self-evaluation questionnaire [27]	Nil

The patient-level clinical trial outcomes include all the outcome measures that would be used in the future large-scale implementation trial. They are being collected at baseline and 3 weeks after randomization during phase II. If a patient is discharged before the scheduled 3-week assessment, every attempt will be made to collect all the patient-level measures prior to their discharge. The purposes of collecting these data are to ensure they are all suitable outcome measures for the future large-scale implementation trial and that they are all feasible to collect as well as to estimate the impact of the approach taken to guide future sample size calculations. They include measures of mobility, general health and function, quality of life, and physical activity. In addition, data on falls and adverse events will be collected over the 3-week intervention period (see Table 4).

**Table 4:** List of patient-level clinical trial outcome measures collected during phase II. These data will be collected at baseline (pre) and 3 weeks (post) for all patient-participants

Outcomes	Pre	Post
Mobility		
1. Short Physical Performance Battery (SPPB) continuous score, 0–3 [28]: measures time taken to complete tasks of gait speed, standing balance, and standing up and sitting down; higher score indicates better mobility	Y	Y
2. Short Physical Performance Battery (SPPB) total score, 0–12 and subscales [29–32]; measures mobility; higher score indicates better mobility	Y	Y
3. Single leg stance: measures standing time on most affected leg in 0–10 s; higher scores indicate better balance	Y	Y
4. Maximal balance range test [33]: measures distance reached forward in millimetres; higher score indicate better balance	Y	Y
5. Step test [34]: measures number of steps in 15 s on each leg, higher score indicates better balance	Y	Y
General health and function	Y	N
6. Functional Co-morbidity Index [35]: collects information on 26 comorbidities and any other the patient reports	Y	Y
7. Technology exposure survey: collects number and frequency of usage of digital devices by Patient-participants before and during the trial	Y	Y
8. Activity Measure for Post-Acute Care (AM-PAC) [36] short form, 0–24 points; self-reported measures of basic mobility; higher score indicates more independence		
Quality of life		
9. European quality of life 5-dimension subscale score (EQ5D-5L) [37], 0–5 points; consists of the EQ-5D descriptive system that comprises 5 dimensions of health and the EQ visual analogue scale (EQ VAS) which records the patient’s self-rated health on a 0–100 visual analogue scale; higher score indicates better quality of life	Y	Y
Physical activity		
10. Average steps per day measured over a 3-day period (1 day at baseline) using the StepWatch activity monitor [38]; higher stepcount indicates greater physical activity	Y	Y
Falls		
11. Falls and fall related injuries from hospital incident reporting system: number of falls	N	Y
Adverse events (intervention group only)		
12. Number of adverse events and details. An adverse event (minor and serious) is defined as an unwanted and usually harmful outcome occurring while the person is participating in the intervention, i.e., whilst undertaking rehabilitation using digital devices (e.g., a fall while using digital devices with no or minor injury (minor AE) or serious injury such as a fracture ( serious AE).	N	Y

### Primary outcomes

The primary outcome measures are three of the key feasibility outcome measures that are deemed most important for determining the feasibility of conducting a future large-scale implementation trial. They will be collected during phase II from the study records and are described below.

- Management: dosage

Data will be collected to reflect how well the physiotherapists document the dosage of exercises provided both as part of usual care (both groups) and as part of the intervention (exercises prescribed with digital devices to the intervention group). The exercise practice sheets will be audited for both completeness and legibility, and the results presented as percentages.

b. Processes: recruitment rate

This will be measured in terms of number of participants recruited per week (compared to the number screened).

- c.Success of implementation: fidelity

Data will be collected to capture the fidelity of using digital devices to prescribe appropriate exercises. That is, to determine whether the participants engaged in additional repetitious practice and if so, to quantify the amount of time spent in additional repetitious practice. This will be determined by measuring the average total active time in minutes per day using digital devices for mobility training. These data will be captured in the intervention group exercise practice sheets (and the control group practice sheet to check if any contamination has occurred) (see Table 3).

### Secondary outcomes

Secondary outcomes will also guide the design of full trial processes, sample size calculations and outcome choice (by exploring impacts on patient-level and implementation measures) and ways to support the physiotherapists to deliver the intervention (by exploring determinants on implementation success). All secondary outcomes are listed in Tables 3 and 4. Patient-level outcomes will be assessed by a physiotherapist who has been trained in conducting the outcome measures and is blinded to group allocation.

### Data analysis

#### Sample size

##### Phase I

All physiotherapists working in the rehabilitation ward will be recruited as therapist-participants.

##### Phase II

A total of 30 Patient-participants (15 per group) will be recruited. The sample characteristics will be similar to the target population for the future large-scale implementation trial. We reasoned that this sample size would provide sufficient information to deem feasibility [21].

### Data management and analysis

Feasibility trial process measures and implementation outcomes will be collected using Excel spreadsheets and through other study documents (e.g., practice sheets). Patient-level data and implementation determinants questionnaire data will be entered into a study REDCap database with license held by the University of Sydney. Data analysis will be conducted using STATA statistical software. Most feasibility outcome measures will be descriptively presented using frequencies, total numbers, and percentages or proportions. The exception is the patient-level clinical trial outcomes and any feasibility outcome measures collected for both groups of phase II. These data will be analyzed using the intention-to-treat dataset. That is, all patients randomized into the study irrespective of adherence to interventions. Between-group comparisons for each of the continuously scored patient-level and implementation outcome measures will be made using linear models with baseline scores entered as a covariate. Fall rates in both groups will be descriptively presented.

## Discussion

There is growing evidence to indicate that exercise prescribed using digital devices can improve mobility outcomes in people undertaking rehabilitation when provided in addition to usual care [22]. The AMOUNT rehabilitation trial provided further rigorous evidence of effectiveness for a tailored approach to selecting the most appropriate digital device and games to address patients’ mobility limitations while respecting their preferences and goals [9]. However, this intervention was delivered by research physiotherapists employed by study funds. It is yet to be determined if a similar tailored digital intervention can be delivered as an additional dose of exercise by physiotherapists employed in a public hospital with the usual staff available, and whether similar outcomes can be achieved. This feasibility study is the essential ground work recommended to inform the conduct of large-scale implementation trial to answer these question [11].

It is important to undertake implementation research to speed up the translation of research findings into practice. One estimate suggests that is takes 17 years to implement research into clinical practice [23]. Implementing evidence into clinical practice is challenging and can be influenced by factors such as lack of knowledge, skills and resources, competing demands of the clinician, and priorities of the service [24]. The need to test the feasibility of implementation strategies and understand the influences on implementation on a smaller scale first is crucial for determining potential for broader implementation and scale-up in the future [11]. This feasibility trial will therefore provide vital information about the feasibility of running a large-scale implementation trial of digital devices across rehabilitation services in other publicly funded hospitals.

A potential limitation of this study is the risk of contamination between control and intervention participants as the physiotherapists who receive the implementation strategies may inadvertently incorporate the digital devices into their therapy sessions for the participants in the control group. To address and monitor this risk, we will educate the physiotherapists on the risk of contamination and monitor the exercise practice sheets from both groups to ensure that the digital devices are only being prescribed to participants in the intervention group. A cluster or step-wedged trial design may be preferable for the future implementation trial to address this limitation [25]. Another potential limitation is the feasibility of delivering the education and training implementation strategies required to a team with staff who rotate every 3 months and have 5-week student placements. To address this, a physiotherapist employed in the rehabilitation team will work as the clinical champion on this study and provide ongoing training to new staff and students as required. In addition, all education sessions will be recorded so new staff and students can view them at their convenience. A final potential limitation is the requirement of accurate recording of dosage data for both groups in the study, with dosage of practice identified as the likely primary implementation outcome for the future large-scale implementation trial. To address this, procedures for recording dosage have been created. We will audit and provide feedback to physiotherapists on their documentation and recording of dosage in the exercises practice sheets. In addition, recording of dosage will be evaluated as a primary outcome of this feasibility study.

A strength of this study is that the protocol has been developed considering current best practice guidance in both feasibility and implementation fields [11, 26]. The use of the hybrid design has the advantage that it will test both implementation and intervention in the same trial, which reduces the time required to test both separately and accelerates the implementation process [10]. This trial will provide important information about the feasibility of physiotherapists implementing exercises using digital devices in a busy public hospital setting. It will also provide important information on the suitability of the implementation strategies to train and support clinical physiotherapists to embed digital devices in a rehabilitation setting. This study is the critical first step to the design and future conduct of a large clinical trial to implement exercises using digital devices in rehabilitation.

## Supplementary Information

**Additional file 1.** SPIRIT Checklist. Filled in SPIRIT Checklist for manuscript submission.

**Additional file 2.** Intervention protocol. Document guiding decision of digital device selection for intervention.

**Additional file 3.** Description of digital devices used in the trial for intervention participants. Name of digital devices used and their description. participants.

**Additional file 4.**Therapist-participant questionnaire to explore determinants of implementation success. Questionnaire used to capture the physiotherapists change in capability, opportunity, and motivation to prescribing and delivering rehabilitation using digital devices, developed based upon the COM-B self-evaluation questionnaire.

## Data Availability

De-identified patient-level data will be available to other researchers on request to Chief Investigator when the main study results are published.

## Abbreviations

AMOUNT: Activity and MObility UsiNg Technology
COM-B: Capabilities, Opportunities, Motivation-Behaviour
ANZCTR: Australian and New Zealand Clinical Trial Registry
COVID-19: COronaVIrus Disease of 2019
USA: United States of America
LLC: Limited Liability Company
CONSORT: CONsolidated Standards Of Reporting Trials
SPIRIT: Standard Protocol Items: Recommendations for Interventional Trials
TIDieR: Template for Intervention Description and Replication
SPPB: Short Physical Performance Battery
MMSE: Mini-Mental State Examination
MoCA: Montreal Cognitive Assessment
REDCap: Research Electronic Data Capture
ERIC: Expert Recommendations for Implementing Change
