# Supplementary material for: Implementing digital devices to increase mobility training for people receiving inpatient rehabilitation: protocol for a feasibility hybrid type II randomized controlled trial
Source: Pilot Feasibility Stud. 2023 Apr 25;9:69. doi: 10.1186/s40814-023-01298-y (PMC10126551; doi:10.1186/s40814-023-01298-y)
Supplement: Supplementary file 2 — Additional file 2. Intervention protocol. Document guiding decision of digital device selection for intervention. [file 40814_2023_1298_MOESM2_ESM.pdf]

h

Activity and MObility UsiNg Technology rehabilitation trial

# PHASE 2 INTERVENTION PROTOCOL

THE UNIVERSITY OF SYDNEY

Institute for Musculoskeletal Health
School of Public Health
Faculty of Medicine and Health

NSW GOVERNMENT Health
South Eastern Sydney Local Health District

THE PRINCE OF WALES HOSPITAL

# Contents


Table of Contents

INTERVENTION PROTOCOL ........ 2

Aim ........ 2

Setting ........ 2

Length of Program ........ 2

Provider of intervention ........ 2

Frequency and Duration ........ 2

Intensity ........ 2

Type ........ 2

Supervision and safety ........ 3

Progression ........ 3

Feedback ........ 3

Goal setting ........ 4

MOBILITY LIMITATIONS TABLES ........ 9

Standing Up from a chair ........ 9

Changing directions while walking ........ 15

DEVICES ........ 18

NINTENDO WII ........ 18

INTENDO WII U ........ 27

XBOX KINECT ........ 38

HUMAC 2013 (v.150) ........ 44

FYSIOGAMING 2015 (v2.1) ........ 55

LusioMATE ........ 62

PTX – PhysioTherapyeXercises ........ 75

CLOCK YOURSELF ........ 82

STEPWATCH ........ 84

INPATIENT PRACTICE SHEET ........ 87


INPATIENT PRACTICE SHEET ........ 87

# INTERVENTION PROTOCOL

## *Aim*

To increase mobility and physical activity through the addition of tailored prescription of affordable technologies in addition to usual care for people admitted to the General Rehabilitation ward of Prince of Wales Hospital with decreased mobility.

## *Setting*

The inpatient intervention will be conducted within the General Rehabilitation ward and gym of the Prince of Wales Hospital.

## *Length of Program*

Inpatient program for 3 weeks after enrolment.

## *Provider of intervention*

The physiotherapist will determine the most appropriate intervention based on the baseline assessment, participant goals, and the digital devices suitable in accordance with the intervention protocol. A physiotherapist will provide the inpatient intervention one-on-one for the length of their inpatient rehabilitation. A trained physiotherapy assistant, physiotherapy student or a carer may also provide the inpatient intervention one-on-one under the supervision of a physiotherapist. If the participant is deemed suitable for safe supervised practice at or during the length of study, with or without environmental set up, then the participant will be trained accordingly.

## *Frequency and Duration*

Participants should participate in an interventions of 30-60 minutes per day using digital devices for
≥5 days per week for 3 weeks after enrolment into the study.

## *Intensity*

Nil specified, as per participant's ability.

## *Type*

The prescription of technology to target mobility and physical activity problems will include, but will not be limited to, the use of video, computer and tablet programs and applications to encourage structured exercise and other forms of physical activity as well as

pedometers to provide feedback on activity levels. The chosen technologies are all relatively low cost and will provide feedback on mobility task or physical activity performance or dose. The physiotherapist will choose the most appropriate technology/ies for an individual participant by following this protocol which has been refined through use in the first phase of this trial, the AMOUNT trial. The research protocol includes tables to guide the choice of exercises/games and technology based on mobility limitations, and tables detailing all the different games/exercises within each device and how to utilise them for various mobility limitations.

Each technology to be used will 1) provide feedback about task performance; 2) facilitate individualised tailoring and progression of exercise or physical activity; 3) enable progress towards a functionally relevant goal(s) to be recorded and reviewed; and 4) is relatively inexpensive.

## *Supervision and safety*

The safety of the participants is of the upmost importance. Exercises should be challenging (e.g. no hand support where possible), but the environment or supervision used to maximise safety. For example, the participant can be set up next to a wall with a table or chair on their other side to increase safety while practicing standing activities. The physiotherapist will provide one-on-one supervision initially to ensure safety and to help participants to use the digital devices outside of therapy times. Participants will be encouraged to exercise unsupervised or with family/friends once they are safe to do so, however the physiotherapist will still oversee the sessions to ensure the prescribed dose is delivered.

## *Progression*

- Reduce length/frequency of rest period between exercises
- Reduce *sitting* rest periods (i.e., stay standing between exercises)
- Increase difficulty of mobility task
- Increase difficulty of chosen technology (e.g., games/exercises that require timing, decisions, UL use)
- Progress type of mobility task (e.g., from standing to stepping exercises)
- Increase participant independence with exercises and use of technology

## *Feedback*

As stated above, each device must be able to provide feedback about task performance. Feedback can be provided as Knowledge of Results (KOR) or Knowledge of Performance (KOP).

KOR is a form of augmented feedback where verbal, or visual information is given to a participant at the end of the performance of a skill; the feedback is about the outcome of the performance, rather than about the movements which brought about the performance. KOP is a form of augmented feedback given verbally or visually either during or after the performance of a skill.

The feedback contains information about the nature of the movement pattern produced during the performance and may include identification of the parts of the skill which were performed correctly, and the parts performed incorrectly.

KOR is provided visually from the devices such as the score in completing a game or exercise (Wii, Xbox, Humac games, Fysiogaming) or the distance walked (phone app) or steps taken (Fitbit), or the number of repetitions achieved (iPad apps). Some devices also provide KOP visually such as the location of centre of pressure or centre of mass (e.g., ski slalom exercise on the Wii, Humac exercises, Stepping Tiles).

The Physiotherapist can also provide feedback verbally in the form of KOP (e.g., kinematics of performing an activity such as standing up) or KOR (e.g., number of repetitions, duration of an exercise).

Many of the devices also provide the option for KOP or KOR to be graphed or displayed in a table which can be provided to the participant.

## *Goal setting*

Goal setting is a critical element of the intervention protocol. One of the features of the technologies used in the trial is that they enable progress towards a functionally relevant goal which is to be recorded, reviewed and can be graphed. Goals play a number of important roles in rehabilitation. For our participants, they can provide an agreement and structure of what they are going to work towards, and motivation to strive for achievement. By the use of linking sub-goals to global goals, they can also help the participant understand how the exercises/technologies are helping them work towards their more meaningful participation goal. For staff, goals can provide a framework and timeline for the intervention provided, and to ensure that they progress the participant when appropriate throughout the intervention. They may also provide valuable process information to help describe the intervention we delivered and the success of the participants within the intervention.

Goal setting is a skill that develops with practice. There are a few key elements to writing a good goal and can be remembered by the term “SMART” goals. That is

**S** specific
**M** measurable
**A** attainable- yet still challenging
**R** relevant to stakeholders – the participant, physiotherapist, family etc

**T** time-bound

- Specific: The goal must be based around a specific observable behaviour or activity. For example, standing up, walking, stepping, reaching, boarding a bus, grocery shopping. Other elements that can make your goal very specific include defining the conditions that are required for performing the goal i.e. how will the client perform the task – with use of equipment, assistance, independently, verbal cues, supervision; and defining where the goal activity will be performed i.e. location, environment. For example: Jack will walk independently from his home to the local shops using a walking stick.

- Measurable: Need to be able to objectively measure whether the goal is achieved. May include 2 criteria: how achievement will be measured (e.g. test or piece of technology/game) and criteria for acceptable standard of outcome performance (e.g. 90% accuracy, < 5 mins, 8 out of 10 repetitions). (Mogensen 2008). For example, at least 6000 steps 3 days per week using the Fitbit.

- Attainable: Goals should be realistic yet challenging. Research in people who have had a brain injury tell us that specific difficult goals results in better immediate performance on motor and cognitive tasks than "do your best" type of goals (Levack, 2006). It can be challenging in itself to set challenging goals, as a rule of thumb, Short Term Goals are "probably attainable" and Long-Term Goals are "possibly attainable" (Playford 2009).
- Relevant: Goals should be relevant to all stakeholders, in this instance; the stakeholders are the participants, perhaps family/friends of the participant, and us as staff on the trial. For the participant, this means involving them in the goal setting process and understanding what types of activities/roles they want to return to. For us, this means incorporating goals that work in some way towards improving mobility and physical activity. Non-collaborative goal setting (that is, the therapist independently determining all the goals without any discussion with the patient has been cited as one of the reasons for failure in neurorehabilitation (van der Broek 2005). Collaborative goal setting can be challenging with some people who may have unrealistic goals or who may have never set a goal before in their life. How involved the participant is and how important goal setting is to motivate the participant will probably vary considerably between participants.

- Timebound: The goal should incorporate a timeframe that you expect the goal to be achieved in. Not all goals have to be set for the same period of time; you just need to be able to review each goal within the timeframe for which it was set. If a goal is not achieved in that time, it should be rated as to how much achievement has been attained in that timeframe. No goal should be rated as ongoing, a new goal should be written.

## Global goals and sub-goals

One way to incorporate relevant goals for someone early in the intervention phase that still needs to do lots of practice at the activity or impairment level is to set an overarching global goal at the participation level. This is a goal that the patient is aiming to achieve at some

time in the future. It may be getting back to living in their home independently or being able to walk to the local shops to do their groceries or go to the park with their grandchildren. This type of goal is a long-term goal and is likely to take more than 6 weeks to achieve (perhaps even the whole of the intervention phase). You can then set smaller sub-goals under this larger goal that will help the participant work towards their more meaningful long-term goal. Below is an example of global goals with sub-goals. In some cases, intervention may be focused on improving a large task that may take a significant amount of time. In these cases, global goals may be written, with appropriate sub goals that break this large task down into more achievable steps. The timeframe for this global goal may be for a number of months with the sub-goals underneath changing as each are achieved and the client moves closer to achieving their global goal.

## Goal rating

A 5-point rating scale for the achievement of goals will be used to rate the achievement of goals by participants in the intervention group. The five rating choices are:

1. Not achieved
2. Partially achieved
3. Mostly achieved
4. Achieved
5. Achieved +

**1. Not achieved:** The participant did not successfully achieve any component of the goal due to for example, the technology was not provided, they did not practice, they no longer agreed to the goal, they tried but no progress was achieved, the goal was set too difficult.

**2. Partially achieved** (1-49%): The goal was partially achieved/completed, but less than 50%. For example, if there is a qualitative and a quantitative component of the goal, the person may have achieved one component, but was nowhere near achieving the other component. Another example may be that they may have started achieving the goal but dropped off and were not consistent. Another example may be a number of components may be included in a goal and some are achieved or completed, but less than 50% of them.

**3. Mostly achieved** (50-94%): The goal or plan has been mostly achieved but less than 95%. For example, if there is a qualitative and a quantitative component of the goal, the person may have achieved one component, and almost achieved the other component. Another example may be where a quota or set number of sessions or exercises are set as the goal. They may have come close to achieving this goal but did not quite do all that was required. For example, they may have gone for a walk 2x a week where the goal was set at 3x a week. Another example may be a number of components may be included in a goal or plan, where more than 50% are achieved but less than

95%

**4. Achieved (95-105%):** The goal set is achieved.

**5. Achieved + (> 105%):** This is awarded when a participant well exceeds the expectation of the goal that was set. For example, the participant is consistently completing >2,000 steps per day using Fitbit, where the goal was only set for 3x per week for > 1,500 steps. Another example for this rating may be that they achieve a goal much earlier than the timeframe that was set.

Next to each rating there will be room to make a comment about the participant's progress of each goal. This allows you to provide quantitative or qualitative information about the progress of the participant for each goal.

## Goal example

An example of a global goal and sub-goals for the AMOUNT intervention: <u>Global goal:</u>

To walk independently to and from Randwick metro station to home (~1km away) at least once per week to access community group activities within 5 months.

<u>Subgoals:</u>

- To complete all your IPad PTX walking program exercises at least 5 times per week for the next 4 weeks.
- To walk to the end of your street and back without stopping on at least 3 days of the week measuring your walk using Fitbit for the next 2 weeks.
- To walk at least 6,000 steps on at least 3 days of the week measured using your Fitbit for the next 2 weeks.
- To play the WiiFit jogging plus game at least 3 times a week and complete the track in <8 minutes within 4 weeks.
- To achieve a score of >......... on the Xbox Kinect "stomp it" game by stepping only on the purple and orange lights within 2 weeks.
- To complete 50 repetitions without a rest using the Humac left right weight shift game in <5 minutes with a score > 80% within 1 week.

- To achieve a score of >........on the Fysiogaming sideways walking game (difficulty level 5) within 2 weeks.
- To complete 50 stepping exercises on the stepping tiles/Clockface exercise with your right foot without using your hands within 2 weeks

•

# MOBILITY LIMITATIONS TABLES

## *Standing Up from a chair*

| Adaptive strategy/problem | Set-up | Easy games/exercises | Medium games/exercises | Hard games/exercises |
|---|---|---|---|---|
| **Weight borne principally through intact side** | Intact leg forward, on block, height of chair | **Humac** Weight bearing (“>” mode); Force vs. Time<br>**Fysiogaming** sit to stand (level 1-10); Assessment Centre<br><br>**Lusio:** STS using easy level of Jumpy rabbit, Basketball, Flying rocket, and Steelman<br>**iPad App: PTX** preparation for standing up (low difficulty) | **Humac** Weight bearing (“>” mode), Force vs. Time<br>**Fysiogaming** sit to stand (level 11-20)<br>**iPad App: PTX** standing up (medium difficulty)<br>**Lusio:** STS using medium level of Jumpy rabbit, Basketball, and Steel man<br>**iPad App: PTX** standing up (medium difficulty) | **Fysiogaming** sit to stand (level 21-30 or dynamic mode)<br>**iPad App: PTX** standing up (high difficulty)<br><br>**Lusio:** STS using hard levels of Jumpy rabbit, Basketball |
| **Wide base of support** | Restrict BOS markers/blocks on ground, height of chair | **Fysiogaming** sit to stand (level 1-10); Assessment Centre<br>**iPad App: PTX** standing up (low difficulty) | **Fysiogaming** sit to stand (11-20)<br>**iPad App: PTX** standing up (medium difficulty) | **Fysiogaming** sit to stand (21-30 or dynamic mode)<br>**iPad App: PTX** standing up (high difficulty) |
| **Falls backwards** | Height of chair | **Humac** Force vs. Time; scale; luge<br>**Fysiogaming** sit to stand (level 1-10); Assessment Centre<br><br>**WiiUFit:** Core luge<br>**iPad App: PTX** standing up (low difficulty)<br>**Lusio:** STS using easy level of Jumpy rabbit, Basketball, Flying rocket, and Steelman | **Humac** as for easy but progress difficulty level, boundaries of movement<br>**Fysiogaming** sit to stand (11-20)<br>**iPad App:PTX** standing up (medium difficulty)<br><br>**Lusio:** STS training using medium level of Jumpy rabbit, Basketball, | **Fysiogaming** sit to stand (21-30 or dynamic mode)<br>**iPad App: PTX** standing up (high difficulty)<br><br>**Lusio:** STS training using hard level of Jumpy rabbit, Basketball, Music smash |

| | | | | |
|---|---|---|---|---|
| | | | Music smash | |
| **Reduced speed of movement** | Height of chair | **Humac** Force vs. Time; scale; luge<br>**Fysiogaming** sit to stand (level 1-10); Assessment Centre<br><br>**Lusio:** STS using easy level of Jumpy rabbit, Basketball, Flying rocket, and Steelman<br>**iPad App:** PTX standing up program | **Humac** as for easy but progress difficulty level, boundaries of movement<br>**Fysiogaming** sit to stand (11-20)<br>**Lusio:** STS training using medium level of Jumpy rabbit, Basketball, Music smash<br>**iPad App:** PTX standing up | **Fysiogaming** sit to stand (21-30)<br>**iPad App: PTX** standing up; high difficulty<br><br>**Lusio:** STS training using hard level of Jumpy rabbit, Basketball, and Music smash |

## *Maintaining a standing position*

| Adaptive strategy/problem | Set-up | Easy games/exercises | Medium games/exercises | Hard games/exercises |
|---|---|---|---|---|
| **Decrease ability to stand EWB** | BOS, environment set up (wall, table, chair, supervision) | **Humac:** CTSIB,Weightbearing; Weightbearing XY; Centre of Pressure<br>**Stepping Tiles:** standing EWB<br>**iPad APP: PTX** Maintaining standing | **Humac:** as for easy but progress difficulty level, boundaries of movement | **Humac:** as for easy & medium but progress difficulty level, boundaries of movement |
| **Holding self stiff (avoids threats to balance)** | BOS, environment set up (wall, table, chair, supervision) | **Humac:** Stability; Mobility; Stability envelope; Limits of Stability Wightbearing XY,Targets, Random Motion;<br>**iPad APP: PTX** Maintaining standing | **WiiFit:** Table tilt; heading; balance bubble; hula hoop; ski slalom; snowboard slalom; Perfect 10; Skateboard arena; snowball fight (modified)<br>**Xbox:** wall breaker; Fruit Ninja<br>**Humac:** as for easy but progress difficulty level & boundaries of movement; Roadway; Balance; Ski; Snowboard; Luge; Flight | **WiiFit:** tightrope; driving range; snowball fight<br>**Humac:** as for easy & medium but progress difficulty level & boundaries of movement; Pong; Breakout |

| | | | | |
|---|---|---|---|---|
| **Decrease loading the affected leg** | BOS, environment set up (wall, table, chair, supervision) | **Humac:** Weightshift; Targets; Weightbearing XY<br>**WiiFit:** Penguin slide (modified), Big Top Juggling (modified)<br>**Stepping Tiles:** reaching in sitting, hip extension over the side of the bed, Shifting weight in standing<br>**iPad APP: PTX** Maintaining standing; | **WiiFit:** Table tilt, heading, balance bubble, ski slalom, ski jump, snowboard slalom, Perfect 10, Skateboard arena, hula hoop, tightrope (modified), Penguin slide, Tilt city (modified)<br>**Humac:** as for easy but progress difficulty level & boundaries of movement; Roadway ML; Balance; Ski; Snowboard; Luge; Flight<br>**Lusio:** Weight shift in standing medium levels of music smash, flying rocket, hockey | **WiiFit:** Ski jump; tightrope; driving range; snowball fight; Tilt city; Big Top juggling<br>**Humac:** as for easy & medium but progress difficulty level & boundaries of movement; Pong ML; Breakout ML; Roadway<br>**Lusio:** Weight shift in standing hard levels of poppop, music smash, hockey |

## *Reaching while Standing*

| Adaptive strategy/problem | Set-up | Easy games/exercises | Medium games/exercises | Hard games/exercises |
|---|---|---|---|---|
| **Flexes at hips and/or protracts shoulder instead DF at ankles when reaching forward** | Set targets to reach for Fysiogaming, environment set up (wall, table, chair, supervision) | **Humac:** Weight bearing (^);<br>**Fysiogaming:** leaning trunk forward and backward; all sides (level 1-10); Reaching (level 1-10)<br>**iPad APP:** PTX Reaching in standing exercise<br><br>**Lusio:** Easy levels of road bomber, flying rocket, music smash, jumpy rabbit, basketball | **Humac:** as for easy but progress difficulty level & boundaries of movement<br>**Xbox:** 20,000 leaks; wall breaker<br>**Fysiogaming:** leaning trunk forward and backward; all sides (level 11-20); Reaching (level 11-20)<br>**Lusio:** Medium levels of music smash, jumpy rabbit, basketball, and steel man | **Humac:** as for easy & medium but progress difficulty level & boundaries of movement<br>**Fysiogaming:** leaning trunk forward and backward; all sides (level 21-30); Reaching (level 21-30)<br>**Lusio:** Hard levels music smash, jumpy rabbit, basketball, sliding santa, and steel man |

| | | | | |
|---|---|---|---|---|
| **Moving trunk instead of moving at hips and ankles when reaching sideways** | Set targets to reach for Fysiogaming, environment set up (wall, table, chair, supervision) | **Humac:** Weight shift<br>**Fysiogaming:** leaning trunk to the sides, all sides (level 1-10)<br>**iPad APP: PTX** Reaching in standing exercise<br><br>**Lusio:** Easy levels of road bomber, flying rocket, music smash, jumpy rabbit, basketball | **Humac:** as for easy but progress difficulty level & boundaries of movement<br>**WiiFit:** snowboard slalom, Wii hula hoop<br>**Fysiogaming:** leaning trunk to the sides, all sides (level 11-20)<br>**Lusio:** Medium levels of music smash, jumpy rabbit, basketball, and steel man | **Humac:** as for easy & medium but progress difficulty level & boundaries of movement<br>**Fysiogaming:** leaning trunk to the sides, all sides (level 21-30)<br>**Lusio:** Hard levels music smash, jumpy rabbit, basketball, sliding santa, and steel man |

## *Stepping while standing*

| Adaptive strategy/problem | Set-up | Easy games/exercises | Medium games/exercises | Hard games/exercises |
|---|---|---|---|---|
| **Decrease speed of stepping** | environment set up (wall, table, chair, supervision) | **Fysiogaming:** walking sideways (level 1-10); lunges; dynamic balance (level 1-10); walk in place (level 1-10)<br>**iPad APP: PTX** Stepping in standing exercise<br>**Lusio:** Stepping forward, sidewards, backwards, toe taps using easy levels of road bomber, flying rocket, jumpy rabbit, basketbal<br>**Clock Yourself App**: Stepping using simple colours | **WiiFit:** jogging plus; step basic (modified)<br>**WiiFamily Trainer:** stone stepper, sprint challenge<br>**Xbox:** 20,000 leaks; space pop; stomp it (modified); run the world<br>**Fysiogaming:** walking sideways (level 11-20); dynamic balance (level 11-20); walk in place (level 11-20)<br>**Lusio:** Stepping forward, sidewards, backwards, toe taps using medium levels jumpy rabbit, basketball, steel man, music smash<br>**Clock Yourself App**: Stepping using simple clock. Increase | **WiiFit:** Cycling; step basic;<br>**WiiUFit:** hosedown, dessert, obstacle course, mole stomper<br>**Xbox:** river rush; rally ball; reflex ridge; stomp it;<br>**Fysiogaming:** walking sideways (level 21-30); dynamic balance (level 21-30); walk in place (level 21-30)<br>**Lusio:** Stepping forward, sidewards, backwards, toe taps using hard levels of jumpy rabbit, basketball, steel man, sliding santa<br>**Clock Yourself App**: Stepping using simple |

| | | | | |
|---|---|---|---|---|
| | | | stepping speed in options<br>**iPad APP: PTX** Timed stepping forward, sidewards, toe taps, step up forwards and sidewards ect in standing exercise for eg how many in one minute | clock, brain games, coordination, complex combinations, athletic agility. Increase the speed in options. |
| **Increase time spent in double support** | environment set up (wall, table, chair, supervision), | **Fysiogaming:** side stepping; side strides; lunges; hip abduction<br>**Lusio:** Stepping forward, sidewards, backwards, toe taps using easy levels of road bomber, flying rocket, jumpy rabbit, basketball<br>**Clock Yourself App**: Stepping forward using simple colours, simple clock<br>**iPad APP: PTX** Stepping forward, sidewards, toe taps, step ups forwards and sidewards ect in standing exercise with metronome | **WiiFit:** tightrope (modified)<br>**WiiFamily Trainer:** stone stepper, sprint challenge, mine cart adventure (modified)<br>**Xbox:** juggle it; kick it<br>**Lusio:** Stepping forward, sidewards, backwards, toe taps using medium levels of jumpy rabbit, basketball, steel man, music smash, mountain bicycle<br>**Clock Yourself App**: Stepping forward using simple clock. Increase the speed in options.<br>**iPad APP: PTX** Stepping forward, sidewards, toe taps, step up forwards and sidewards in standing exercises | **Fysiogaming:** hip abduction<br>**WiiFit:** tightrope<br>**WiiFamilyTrainer:** BMX speed, mine cart adventure<br>**WiiUFit:** hosedown, dessert, obstacle course, mole stomper<br>**Xbox:** stack them up<br>**Lusio:** Stepping forward, sidewards, backwards, toe taps using hard levels of jumpy rabbit, basketball, sliding santa<br>**Clock Yourself App**: Stepping using simple colours, simple clock, coordination, complex combinations, athletic agility. Increase the speed in options.<br>**iPad APP:** Increase the distance with stepping, height of block with toe taps and step up exercises |

| | | | | |
|---|---|---|---|---|
| **Short step length** | Line/target to step to, environment set up (wall, table, chair, supervision) | **Fysiogaming:** dynamic balance (level 1-10); side stepping; side strides; lunges<br>**Lusio:** Stepping forward, sidewards, backwards using easy levels of road bomber, flying rocket, jumpy rabbit, basketball<br>**Clock Yourself App**: Stepping forward using simple colours, simple clock<br>**iPad APP: PTX** Increase the distance while stepping forward, sidewards in standing exercises | **Xbox:** 20,000 leaks; stomp it (modified)<br>**Fysiogaming:** dynamic balance (level 11-20)<br>**Lusio:** Stepping forward, sidewards, backwards using medium levels of jumpy rabbit, basketball, steel man, music mash<br>**Clock Yourself App**: Increase the distance while doing stepping forward using simple colours, simple clock<br>**iPad APP: PTX** Increase the distance while stepping forwards, sideways and backwards in standing exercises | **Xbox:** stomp it<br>**Fysiogaming:** dynamic balance (level 21-30); hip abduction<br>**Lusio:** Stepping forward, sidewards, backwards, toe taps using hard levels of jumpy rabbit, basketball, steel man, music smash<br>**Clock Yourself App**: Stepping using simple clock, brain games, coordination, complex combinations, athletic agility. Increase the speed in options. |
| **Trunk inclined forward when stepping with the unaffected leg** | Visual cues to maintain trunk extension; environment set up (wall, table, chair, supervision) | **Lusio:** Stepping forward, sidewards, backwards,,toe taps using easy, levels of road bomber, flying rocket, jumpy rabbit, basketball<br>**Clock Yourself App**: Stepping forward using simple, colours, simple clock<br>**iPad APP:** PTX Stepping forward, sidewards, toe taps, and step ups forwards | **Lusio:** Stepping forward, sidewards, backwards, toe taps using medium levels of road bomber, flying rocket,<br>jumpy rabbit, basketball<br>**Clock Yourself App:** Stepping forward using simple colours, simple clock | **Lusio:** Stepping forward, sidewards, backwards, toe taps using hard levels of jumpy rabbit, basketball, steel man, music smash<br>**Clock Yourself App:** Stepping using simple clock, brain games, coordination, complex combinations, athletic agility |
| **Increased hip circumduction in swing** | Environment set up (wall/block to restrict unwanted movement, block | **Fysiogaming:** knee and hip flexion, knee flexion (level 1-10, 50% ROM) iPad APP: PTX Stepping in standing program | **Fysiogaming:** knee and hip flexion, knee flexion (level 1- 10, 100% ROM)<br>**iPad APP**: PTX Stepping in | **Fysiogaming:** knee and hip flexion, knee flexion (level 11-30, 100% ROM)<br>**Lusio:** Stepping forward, |

| | | | | |
|---|---|---|---|---|
| | height) | **Lusio:** Stepping forward, toe taps and step ups using easy levels of road bomber, flying rocket, jumpy rabbit, basketball<br>**iPad APP:** PTX Stepping forward, toe taps in standing balance | standing program<br>**WiiFamilyTrainer:** stone stepper modified<br>**Lusio:** Stepping forward, toe taps and step ups using medium levels of road bomber, jumpy rabbit, basketball, steel man<br>**iPad APP:** PTX Stepping forward, toe taps in standing balance | toe taps and step ups using hard levels of road bomber, jumpy rabbit, basketball, steel man |

## *Changing directions while walking*

| Adaptive strategy/problem | Set-up | Easy games/exercises | Medium games/exercises | Hard games/exercises |
|---|---|---|---|---|
| **Decrease speed changing direction** | environment set up (wall, table, chair, supervision) | **Fysiogaming:** walking sideways (level 1-10), walk in place (level 1-10)<br>**iPad APP: PTX** changing directions while walking exercises<br>**Lusio:** walking exercises using easy level of red runner, mountain bicycle | **WiiFit:** step basic (modified)<br>**WiiFamilyTrainer:** mole stomper<br>**Xbox:** 20,000 leaks;<br>**Fysiogaming:** walking sideways (level 11-20), walk in place (level 11-20)<br>**iPad APP: PTX** changing directions while walking Exercises<br>**Lusio:** walking exercises using medium level of red runner, mountain Bicycle | **WiiFit:** step basic ;<br>**WiiUFit:** dessert, ultimate obstacle course, scuba search<br>**Fysiogaming:** walking sideways (level 21-30), walk in place (level 21-30)<br>**iPad APP: PTX** changing directions while walking exercises<br>**Lusio:** walking exercises using hard level of red runner, mountain Bicycle |

| | | | | |
|---|---|---|---|---|
| **Shuffling feet to change direction** | environment set up (wall, table, chair, supervision) | **Lusio:** walking exercises using easy level of red runner, mountain bicycle<br>**Clock Yourself App**: Coordination, complex coordination, and Athletic agility at slower speed<br>**iPad APP: PTX** changing directions while walking exercises | **Lusio:** walking exercises using medium level of red runner, mountain Bicycle<br>**Clock Yourself App**: Coordination, complex coordination, and athletic agility at medium speed<br>**iPad APP: PTX** changing directions while walking exercises | **Clock Yourself App**: Coordination, complex coordination, and athletic agility at greater speed<br>**iPad APP: PTX** changing directions while walking exercises |

## *Stairclimbing*

| Adaptive strategy/problem | Set-up | Easy games/exercises | Medium games/exercises | Hard games/exercises |
|---|---|---|---|---|
| **Decreased hip and knee extension when ascending stairs** | Block height, environment set up (wall, table, chair, supervision) | **iPad APP: PTX** Climbing stairs exercise<br>**Lusio:** step ups using easy levels of road bomber, flying rocket, jumpy rabbit, basketball, steel man | **WiiFit:** step basic (modified)<br>**StepWatch** | **iPad APP: PTX** Climbing |
| **Decreased eccentric control knee extensors on decent** | Block height, environment set up (wall, table, chair, supervision) | **iPad APP: PTX** medium difficulty<br>**Lusio:** step downs using easy levels of road bomber, flying rocket, jumpy rabbit, | **iPad APP: PTX** medium difficulty<br>**Lusio:** step ups using medium levels of road bomber, flying | **iPad APP: PTX** high Difficulty<br>**Lusio**: step downs using hard level of flying rocket, jumpy rabbit, basketball, steelman |

| | | |
|---|---|---|
| | basketball, steel man | rocket, jumpy rabbit, basketball, steel man |

## *Physical activity throughout day*

| Adaptive strategy/problem | Easy games/exercises | Devices |
|---|---|---|
| **Prolonged periods of sitting** | Any technology above that the person is standing to participate in | **Lusio:** Set up with any safe standing exercise using Lusio games<br>**Stepwatch:** encourages to take steps<br>**iPhone/ iPad App: PTX** set up to do safe room exercises in standing with or without family throughout the day |
| **Prolonged overall sedentary time** | Any technology above that the person is standing to participate in | **WiiFit:** jogging, step basic modified), free stepping (slow pace)<br>**Lusio:** Set up with any safe standing exercise using Lusio games<br>**Stepwatch:** encourages to take steps<br>**iPhone/ iPad App: PTX** set up to do safe room exercises in standing with or without family throughout the day |
| **Decrease steps throughout the day** | Any technology above that the person is stepping in standing | **WiiFit:** jogging, step basic (modified) free stepping<br>**Lusio:** Set up with any safe stepping exercise using Lusio games<br>**Stepwatch:** encourages to take steps<br>**iPhone/ iPad App: PTX** set up to do safe room stepping exercises in standing with or without family throughout the day |

# DEVICES

## *NINTENDO WII*

### Getting started with Wii

a. Turn on power and load the software WiiFit Plus. You will need a Wiimote, balance board and nunchuk to play all the games in WiiFit Plus (Note: check batteries and ensure you have spares ready to go)
b. You will start at the main menu of Wii. There are a number of different boxes to choose, there are 3 that are relevant: 1) top left-hand corner is the software that you have loaded, select that to play the games. 2) Mii Channel is 2nd top left box with lots of faces, this lets you create a Mii (see below). 3) Envelope down bottom right hand corner, this links you to the calendar which records play time, you can use this to verify play time if participant independent, or to record it even if you are supervising. Select envelope and select calendar, then choose which date you wish to view, it will display different play times (if there has been a break in game play, and total time for that day.

### Creating a new Mii

You could create a Mii for each participant if they want, but otherwise you could perhaps make a male and female and use for all inpatients. Creating their own takes time, but that Mii could top the leader board of games and motivate in that way.

c. Go to Mii channel
d. Select “Start”
e. Choose smiley file icon with + sign.
f. Select male or female
g. You can start from scratch but quicker to select “choose a look-alike”
h. You can modify different features e.g. hair, eye colour etc.
i. Once you have finished making modifications, select “quit” and then “save and quit”.
j. Enter a name
k. Return to the Wii menu (arrow circle top left-hand side)

## Getting started with WiiFit

I. Select WiiFit (at top left-hand corner of Wii menu) and Start
    i. If you haven't put your Mii into WiiFit you need to do this first (you might want to do this before the participant comes) unless you want to show them how to do it. (Note: if you don't want to create a Mii and just want to get started, you can select the trial button (outline of person with ? in middle and select from 6 Mii's they give you).
    ii. The Smiley face with the + sign on the left side lets you add a Mii you have previously created in the Mii Channel.
    iii. Select "create"
    iv. Press the A button to quickly scroll through the balance board talk and select the Mii for the participant you are working with and hit yes.
    v. Add height and DOB for participant
    vi. Press the A button to quickly scroll through the balance board talk and turn on balance board as per picture on screen
    vii. Select "training" to go to the games in WiiFit Plus. The categories are: training plus, yoga, muscle workouts, aerobic exercises, balance games

## Game selection

- Don't start with games that don't allow error (WiiFit Balance: balance bubble and tightrope, zazen), select game where certain level of success can be achieved.
- WiiFit plus training plus games: Rhythm Kung Fu , Segway Circuit, Bird's eye-bull's eye, rhythm parade, table tilt plus, balance bubble plus deemed not appropriate for rehabilitation populations in this trial.
- WiiFitplus balance exercises: balance tests (basic balance test, agility test, stillness test, dual balance test, prediction test, peripheral vision test, judgement test) not appropriate for this trial as only last short time, you cannot select which test to do and there are a lot of screens to be navigated through to complete the short test.

## Getting started with Wii Family Trainer

<u>Family Trainer/Family Trainer Extreme Challenge</u>

i. You will need the Game Mat, and Wiimote to play the games.

ii. After inserting the CD into the Wii Console, with your Wii remote select "family trainer"/"family trainer extreme challenge" (the first box in the top left corner)

a. o select, point towards the item you wish to select on the screen and press “A”

iii. Select “start”

iv. Select “A to start”

v. Mode select screen: select “single player”

vi. Select character

   a. OR “new”

      i. Select face

      ii. Type name

      iii. Select body type

vii. Select “free play”

viii. Choose the game to play (there are down/up arrows on the right side of the screen to scroll)

## Other helpful hints for using the Wii

m. Home button takes you to option to Wii Menu.

n. When in WiiFit game, it is often easier to use arrows than move the Wiimote

| WII FIT | | | | | | | |
|---|---|---|---|---|---|---|---|
| **Mobility activity** | **Game** | **Game length** | **Description** | **Movement/ Feedback** | **Progress/ Motivation** | **Issues/ Additional demands** | **Rehabilitation modifications** |
| **Sitting** | WiiFit balance/ Zazen | Game stops when move | The player is required to sit on the balance board and maintain a quiet seated position so that the candle on the screen does not blow out. | No movement/ Candle flame starts to move if you start moving, does not allow error. | you can maintain position/ time sitting still, time game, leaderboard | Only records when movement occurs, can start unbalanced and move to more balanced position and it will interpret that as negative | Place balance board on plinth with thigh support and feet flat on ground. Start person in balanced position. |
| **Standing** | WiiFit balance/ Heading | ~60secs | The player is required to move weight between their legs on the balance board to 'head' soccer balls being kicked towards them | ML direction / total score at end, allows errors | ↑ target frequency/ leaderboard | Mocking, fast paced/ must miss other objects thrown e.g. shoe | Set small target number of balls to head in timeframe |
| | WiiFit Balance/ Ski slalom | ~60secs | The player is required to ski down a mountain between flags by moving weight between their legs on the balance board | All directions / total score at end, allows errors, | None/ leaderboard | Mocking/ nil | Use dot representing COM rather than person skiing to guide performance |
| | WiiFit Balance/ Ski jump | <60secs 2 jumps | The player is required to stand on balance board and start in a squat position, straighten knees to do a ski jump | COM forward, knee extension / distance jumped, allows error | None/ leaderboard | Mocking/ requires getting timing correct to extend knees | Use dot representing COM rather than person skiing to guide performance |
| | WiiFit Balance/ Table tilt | ≤5mins ↑ time with | The player is required to move their weight on the balance board to guide a | All directions/ ball goes into hole, allows error | ↑ target number / leaderboard, stages achieved | Mocking/ requires timing to get ball in hole | Nil |

| | | | | | | | |
|---|---|---|---|---|---|---|---|
| | | stages | ball(s) into a hole | | | | |
| Standing | WiiFit Balance/* Tightrope tension | ≤ 2mins game stops if fall off | The player is required to step on the spot and move weight (SLS) between their legs on the balance board to walk along the tightrope, semi-squat then extend to avoid objects | ML direction/ does not allow error, fb distance walked before fall, time taken to complete, | In game / leaderboard | Performance was better with SLS | Can perform as step touch exercise to block infront |
| | WiiFit Balance/* Balance bubble | ≤1:30 min game stops if bubble bursts | The player is required to move their weight on the balance board to guide their character along a river without colliding with any objects | Keep COM forward and move ML direction/ does not allow error, fb distance achieved or time to complete | ↓ river width, ↑ choice/ leaderboard | Game can end straight away if hit riverbank/ | Nil |
| | WiiFit Balance/ Penguin slide | ≤ 90 secs | The player is required to move their weight on the balance board to tilt an iceberg upon which a penguin is feeding, to guide the penguin to the fish | ML direction/ score at end, allows error | ↑ target number/ leaderboard, previous score | Mocking/ Requires timing to get fish | Lots of options to modify: slow weight shift each side & fall off each side; balance in middle & therapist direct which way to go, fast shift SLS, step touch to block and count how many times fall off, |
| | WiiFit balance/ Snowboard slalom | ~ 60 secs | The player is required to snowboard down a mountain between flags by moving their weight on the balance board (board side on) | COM (L) leg, AP direction/ score at end, allows error | None/ leaderboard, previous score | Fast paced, difficult to use snowboard feedback | Aim achieve certain number of flag checkpoints |
| | WiiFit training plus/ Perfect 10 | ~ 60secs | The player is required to move towards the numbers to add up to 10 by moving their weight on the balance board | ML and AP directions/ score at end, allows error | ↑ number choices/ leaderboard previous score | Dual task solve maths problem with movement | Nil, can work at own pace. |

| | | | | | | | |
|---|---|---|---|---|---|---|---|
| Standing | WiiFit training plus/ driving range | ≥ 5mins 20 balls to hit | The player is required to swing a golf club by shifting weight between legs on balance board and swinging UL with remote (board side on) | ML direction/ ranks your shots, total score | Difficulty level/ leaderboard | Dual task (Physical + cognitive) swing arm, hold button down and shift weight | Nil, would only use if very keen golfer and interested. |
| | WiiFit training plus/ snow ball fight | ≤90 secs | The player is required to throw snowballs at opponents by moving weight on the balance board and aiming with remote | ML direction/ score at end, allows error | In game, ↑ target frequency/ leaderboard, previous score | Fast paced, requires timing and use of UL with movement | Start with aim to not get hit with snowballs, rather than throw snowballs as well |
| | WiiFit training plus/ Tilt City | ~ 2mins | The player is required to move their weight on the balance board and rotate the Wiimote to guide falling balls into matching colour pipes | ML direction/ score at end, allows error | In game, ↑ target number, frequency | Fast paced, requires timing, use of UL and cognitive decisions for correct colour ball | Choose 1 colour ball to get in correct colour pipe. |
| | WiiFit training plus/ Big Top juggling | 2mins | The player is required to shift their weight in a small range side to side to stay close to the middle on the balance board while juggling balls using the WiiMote and nunchuk | ML direction/ score at end, allows errors | In game, ↑ target number, frequency | Dual task (physical + cognitive) Requires correct timing and use of UL for juggling | Lots of options to modify: can start without juggling and focus on weight shift- slow weight shift each side fast weight shift keep in middle, balance in middle & therapist direct which way to go, SLS, step touch to block, |
| | WiiFit training plus/ skateboard arena | | The player is required to skate through a specific track by moving weight between legs on balance board (board forward) | | | ? May be suitable for younger population | |

| | | | | | | | |
|---|---|---|---|---|---|---|---|
| **Stepping in standing** | WiiFit training plus/ obstacle course | ?? | | | | Fun but difficult | |
| | WiiFit training plus/ jogging plus | 10 mins | The player is required to follow a Mii to complete a jogging track (balance board not required, remote in pocket or hand) | Stepping on the spot/ | ?? | ??Asks 3 questions at end of the surroundings | Can walk on spot not jog, allows you to stop if needed. |
| | WiiFit aerobic exercises/ Hula hoop | ?3mins | The player is required to rotate their hips to keep the hoops spinning, need to lean to one side with arms up to catch new hoops | All directions, ML direction to get hoops/ total score, allows error. | Change to super hula game, ↑ duration and change direction | Timing of leaning to side and lifting arms up to get hoops, not always sensitive to movement. | Just focus on hip rotation, don't try to get new hoops |
| **Stepping in standing, changing directions** | WiiFit aerobic exercises/ Step basic | 3mins | The player is required to step up and down forward and to the sides on the balance board on the coloured footprints on screen which indicate timing and direction | Stepping forward, backward, right and left sides/ ?? , allows error | Change to step plus game, ↑ duration, speed and change of direction | Fast pace, timing + cognitive to step with correct foot, risk of falls, recommend close supervision | Could start with forward steps only, give standing rest when goes sideways |
| | WiiFit aerobic exercises/ Free stepping | Can set time or number of steps | The player is required to step up and down forward and to the sides on the balance board in time with metronome (can watch other programs on TV while doing it) | Stepping forward, backward | In game increase speed of metronome, number of steps, duration | Timing to step with foot, risk of falls, recommend close supervision | Nil |

| WII FAMILY TRAINER | | | | | | | |
|---|---|---|---|---|---|---|---|
| **Mobility activity** | **Game** | **Game length** | **Description** | **Movement/ Feedback** | **Progress/ Motivation** | **Issues/ Additional demands** | **Rehabilitation modifications** |
| **Stepping while standing** | Wii Family Trainer/Stone Stepper | Game finishes when course completed (running timer) | The player is required to stand on the game mat and step on either one of the two middle tiles in order to move jump from pillar to pillar on a straight course while avoiding wobbly red pillars. | Stepping with left or right foot in standing/ character moves forward on screen, time, written feedback (ie. "miss" if fallen off pillar), allows for error | Nil/ leaderboard, completing course in a faster time | Negative feedback at end if record not beat (sad face, "try again") | Instead of standing on the middle two tiles and stepping on the spot, the player can stand on the two arrows pointing down instead and step *forward* onto the middle tiles as required |
| | Wii Family Trainer/Sprint Challenge | Game finishes when the track is finished ("goal" line at end) | The player is required to stand on the game mat and step on the two middle tiles with alternate feet in order to run through a straight course. | Stepping on the spot with alternating feet/character moves forward, speed (mph), time, allows for error | Nil/ leaderboard, completing course in a faster time, increasing speed (mph) | Negative feedback at end if record not beat (sad face, "try again"), character on screen moves quite slow if not stepping fast | Instead of just stepping, the player can march with high knees, using their own hands with elbows held at 90 degrees in front of them as targets for their knees to reach; or, a block can be placed in front of the player to do alternating step taps; or, the player can stand in step stance with one foot on the front arrow and one foot on the back arrow and practice weight shifting while lifting the foot opposite to the weight shift |

| | | | | | | | |
|---|---|---|---|---|---|---|---|
| | ii Family rainer/Mole tomper | ~30 seconds | The player is required to stand on the middle two tiles of the game mat and steps on any one of the 6 arrows in order to stomp on the mole(s) as it pops out of the holes. | Stepping in all directions/hammer hits mole, time, score of moles hit, allows for error | Increase number of moles stomped on/leaderboard | Player may cheat by just using one leg to stomp on any of the arrows/If two moles pop up at the same time this may require jumping – however, not necessary to hit both | Ensure that player is only stepping on the orange arrows with his/her R) foot and the blue arrows with his/her L) foot. |
| | Wii Family Trainer Extreme Challenge/BMX Speed | ~20 seconds | The player is required to stand on the two middle tiles of the game mat and cycle through a track by alternate stepping on the mat and shaking the remote in order to accelerate forwards. | Alternate stepping on the spot/cycle moves forward, speed (mph), time | Nil/leaderboard, speed | Negative feedback at end if record not beaten/Dual UL task, need fast stepping to move more than 5mph, short time of 20seconds | Nil |
| **Stair Climbing** | Wii Family Trainer/Mine Cart Adventure | Game finishes when course is completed | The player is required to stand on the middle tiles of the game mat and steer a cart through the track by lifting one leg up in order to go around bends without falling. Meanwhile, holding the Wii remote horizontally and moving it up and down with two hands to accelerate. | Single leg stance and hole with UL elbow flexion/extension/cart moves forward, time, speed (mph), instructions RE: which leg to lift, allows error (if leg not lifted, cart tips off and game returns to the point in which the cart fell off) | Nil/time, leaderboard | Additional cognitive demands in knowing which leg to lift (with assistance of instructions), dual task of UL to accelerate | Player can play without the UL flexion/extension for simplification – the cart will move slower; a target may be placed (eg. Cup on a table) on the left and right of the player as feedback for leaning from side to side with each bend; a block may be placed in front of the player to do step taps instead of single leg stance. |

# *INTENDO WII U*

## Setting up the Wii U console and GamePad

- Turn on the power via the console and turn the TV to the appropriate HDMI or AV input
- Turn the Wii GamePad on and then sync the gamepad by pressing the red button on the front of the Wii U console and the red button on the back of the gamepad(you will need a pen or something thin to press the sync button on the gamepad)
- Follow the instructions on the gamepad for set-up
- Wii U will automatically configure TV display to TV (thus need to set up on the tv the client will be using)
- Skip set up for TV remote ('Not Now')
- Set up Sensor bar
    - Above or below tv depending on where it is most stable
- Continue through instructions on Gamepad
- Connect to internet
    - If having difficulty connecting to internet, need to manually change settings (see below)
    - http://en-americas-support.nintendo.com/app/answers/detail/a_id/1643/~/how-to- manually-set-up-an-internet-connection
- Follow instructions on GamePad
- GamePad will automatically take you to setting up a new Mii
- Link the Nintendo Network ID or skip
- "Do you wish to automatically receive software from Nintendo via SpotPass"- select Don't Receive

## Introduction to Homepage on GamePad and TV screen

- TV screen and GamePad will show two separate displays of the Homepage, to switch between which one is displayed on the TV or on the Gamepad, press the button in the top right corner or press the X button
    - The display you want is the one with the boxes appearing on the gamepad so you can select the menus. The screen with the many Mii characters is not relevant and thus should not be displayed on the gamepad
- There are a number of different boxes to choose from
    - Top left is the software that you have loaded, select that to play the games
    - Mii Maker is 2$^{nd}$ top left box with lots of faces, this lets you create a Mii (see below)
    - The bottom left box with the green bar graph is the 'Daily Log'. This links you to the calendar which records play time, you can use this

to verify play time if participant independent, or to record it even if you are supervising.

## Getting started with WiiFit Plus U

o. Turn on Wii U console by pressing power on button either on GamePad or Console
p. Insert WiiFit Plus U disc into Wii Console
q. If Mii character has not been made, first create a Mii under the Mii Maker menu (see below) and select this player as the user
r. Select WiiFit Plus U logo (at top left hand corner of Wii menu – image of women doing yoga stance)
s. Wait for system memory to be updated ~2-5 minutes
t. Any previous Wii Fit Plus data will be transferred to Wii Fit U (<1 min)
    i. Can later choose whether to transfer data or start as a new user
u. The Balance Board cartoon will provide instructions on what to do (can either watch on gamepad or on TV). Player must hold gamepad to navigate through menus
v. Follow through with Body Balance Tests
w. Once WiiFit Age has been given, will take player to main screen. Press 'Start'
x. Press 'Training' (see below for navigation through Wii Plaza
y. Press 'Select Exercise'
z. Choose exercise from menu
aa. Sync the WiiMote for easy navigation through menus whilst on balance board – so do not have to hold GamePad (See below)

## Creating a Mii Character

You can create a Mii from the homepage. You could create a Mii for each participant if they want, but otherwise you could perhaps make a male and female and use for all inpatients. Creating their own takes time, but that Mii could top the leader board of games and motivate in that way. You will need both the GamePad and the TV for this.

a. Tap on the Mii Maker
b. Select 'Create Mii'
c. Choose either 'Create Mii from Features' or 'Create Mii From Photo'
d. If Creating from Features
    i. Choose gender
    ii. You can modify different features e.g. hair, eye, colour etc.
    iii. Enter in nickname, birthdate and favourite coloure
    iv. Press 'Save' when finished
e. If creating from Photo

   v. Choose gender, skin tone, eye colour, hair colour, hairstyle
   vi. Take photo with gamepad (camera is on the front of gamepad)
   vii. Select a face that best suits the client/they one they prefer
   viii. Change any features such as hair style, eyebrows, eye style, nose etc.
   ix. Enter in nickname, birthdate and favourite colour.
   x. Press 'Save' when finished

f. Return to homepage by pressing Exit (in bottom left corner of gamepad)
g. Select new player as new user by pressing icon with current user's Mii face. Press switch
h. Press 'add new user'
i. Press ' choose a Mii From Mii Maker'
j. Select user's Mii character and press 'Register'
k. Can/cannot link a Nintendo ID
l. Do not apply parental control
m. Will take you back to main page

## Navigating through main menu

- Body test:
  - In bottom left corner of screen
  - Will take you to body tests (balance)
- Training:
  - In bottom right corner of screen
  - Takes you to games
- In the background there are a number of menus that you can navigate through by swiping left or right over them
  - Album
    - Shows pictures of exercises completed on wii u
  - Notice Board
    - Where your achievements in Wii Fit U are displayed
  - Calendar
    - Can check photos taken by Body Test (only really for weight loss – not relevant for this trial)
    - Can select a day and see the photo, BMI, and CoG results from Body test
  - Graphs
    - Calories burned and distance walked

    - Will see data from Wii Fit U and Wii Fit Meter (steps taken, calories burned)
    - Can change the graph on the left and right side to look at
        - Weight, BMI, Wii Fit Age, Waist size, **Calories burned**, FitCash, **Steps, Distance walked**
    - Can manually enter in data under 'record' icon
- Fit Meter Guide
    - Can use the Fit Meter in the Wii U to measure the calories you burn in daily activities
    - Has an accelerometer and a pressure sensor built in
    - These track METs (intensity of activity) as you move around
    - Records steps, calories burned (includes calories burned going up and down stairs/hills), altitude changes
    - Can send Fit Meter records to Wii Fit U ❼ greater detail on gamepad
        - Can use this data in Wii Fit U to tackle courses from around the world
    - If you don't meet goals for calories during the day, can plan a workout in the Wii Fit U for the remaining calories
    - Can register Fit Meter
    - When syncing the Fit Meter, hold down middle button and aim it at the infrared sensor on the GamePad (black square next to earphone jack)
    - Once you register the Fit Meter the Fit Meter Guide menu will be replaced with "Fit Meter Data" and "Fit Meter Challenge" and a Fit Meter icon will appear next to the Mii character
- Fit Meter icon next to Mii Character
    - Press this when you want to sync data from Fit Meter to gamepad
    - Hold down on middle button on Fit Meter and point at infrared sensor
    - Screen will show how many calories burnt today
        - Can press calorie checker to see how much that equates to for common foods
- Fit Meter Data
    - Displays any information from Fit Meter
    - Eventually data will be over-rided – can save certain sessions by pressing 'lock'. Then can add a comment to it
    - Can change graph from 2 hr to 24 hr period
    - Can swipe right or left across graph to see activity over different hours
    - Different colours correspond to different types of activities
- Fit Meter Challenge
    - Use fit meter data and challenge yourself to complete courses from all round the globe
    - Chose walking or climbing challenge

## Navigating through training menu

- Touch and slide panels on the Wii U GamePad to scroll from left to right
- Tap a panel to select it
- Rankings
  - Can view various rankings to do with training
  - Can see this against all users or just within one user
  - Shows you which games are most played, recently played, calories burned, and time played
- Gym Community
  - Can share training details with other users
  - Need a Nintendo ID for this
- Select Exercise
  - This is where all the exercise games are
  - Choose between Yoga, Muscle, Aerobic, Dance and Balance (total of 74 games)
- Personal Trainer
  - Can create a workout for you based on the calorie goal or exercise time set
  - Can set training type and intensity
  - Can choose based on calorie (gives examples of foods)
    - Can choose type – out of all the exercise menus) or random
    - Can choose intensity (low, medium, high or random)
    - Press 'begin'
  - Based on exercise time
    - Up to 60 minutes
    - Can choose type
    - Can choose intensity
    - Press 'begin'
- Wii Fit U Routines
  - Can choose between lifestyle, health, youth, form and vitality
  - Choose which category you want to address and then choose a subcategory
  - Will choose 3 example exercises and tell you how long it will take and how many calories you will burn
- My Routines

    - Can create a routine by adding items
    - Can choose up to 30 exercises
    - Can select exercise and click on it to change from – explanation to 6 reps

## How to Pair a Wii Remote with the Console

- Press ‘Pair Wii Remote’
- Press the sync button on the controller you want to pair
    - Note: you will need a paper clip or a thin object to be able to push the sync button
    - TV and GamePad display will say whether the pairing was complete
    - Press (B) or Exit

## What to do if having difficulty setting up internet

- First ensure that router is on and that the SSID and password is correct
- If internet is working but the Wii is having trouble connecting to the router you will need to connect to the internet manually
    - You will need a computer to locate the network’s IP address information
        - Windows:
            - Select start button
            - Type CMD and press enter
            - Type ipconfig/all and press enter
            - IP information should be displayed in a list – need to scroll through list and find IP address (or IPv4 address), subnet mask, default gateway and DNS servers
        - Mac
            - Select ‘system preferences’ from apple icon in top left corner
            - Select ‘network’ under ‘internet and network’
            - On the left side, select the type of connection you are using (airport, Ethernet etc. ) the active connections should say ‘connected’
            - Select ‘advanced’
            - Click the TCP/IP button

        - The router's IP address is listed as Router
        - The IPv4 address, subnet mask, default gateway and DNS servers should not be displayed
    - From the Wii U Menu, select 'System settings'
    - Select the 'Internet' icon
    - Tap 'connection types' or press the Y button
    - Select 'manual connection'
    - Enter the following information
        - SSID = the Wi-Fi's network's name (case sensitive)
        - Security: select the encryption method (if you are unsure of what it is, it should say it on the router or go to the computer, look at the wireless network connection, right click and go to properties and it will be under security type
    - Tap IP address and select 'don't auto-obtain' and enter the following information
        - IP address: but add 10 to it. E.g. if the computer's IP address displays 192.168.2.5 you will enter 192.168.2.15
        - Subnet Mask: as displayed when searching for IP address. Often it is 255.255.255.000
        - Gateway: as displayed when searching for IP address
    - Tap confirm
    - An on-screen message will ask you to configure the DNS settings. Tap 'Configure'
    - Tap 'Don't auto-obtain' and enter the follow information
        - Primary DNS: enter 8.8.8.8 then tap 'OK'
        - Secondary DNS: enter 8.8.4.4 then tap 'OK'
    - Tap 'Confirm'
    - Tap 'Save' or press the B button. Tap "save' again
    - Tap 'Connection test' to test the connection
    - If internet is still not connecting, check the password and SSID was entered correctly and ensure the internet is working on another device

## Wii Motion Plus

- Is an expansion device for the Wii Remote video game controlled that allows it to more accurately capture complex motion
- The sensor in the device supplements the accelerometer and sensor bar capabilities of the wii remote to enable actions to be rendered identically on the screen in real time
- The original Wii Remote cannot be used with Wii Play: Motion and other games designed solely for use with Wii MotionPlus technology as such games take advantage of the technology to offer an even greater level of control and precision during gameplay

- Need a WiiMotionPlus™ accessory or a Wii Remote™ Plus which has this new feature built in
- Can use an original Wiimote to replace a nunchuck
- Which games require the Wii Motion Plus Remote
    - Balance Games: Free climbing
    - Aerobic Exercises: Puzzle Squash; Rowing Regatta
    - Dance: All dance games

n. Use the Wii remote to select ‘ TV Only’
o. Select WiiFit (at top left hand corner of Wii Menu) and press ‘Start’
p. Sync the balance board by pressing the red button on the bottom of the balance board and the red button on the console
q. Read instructions on screen and press A to continue

## Other helpful hints for using the Wii U

r. When in WiiFit game, it is often easier to use arrows than move the Wiimote
s. Pressing the Power button on the gamepad will turn the console on – can enable or disable this
t. Buttons generally mean:
    xi. A = enter/continue
    xii. B = back/exit
    xiii. X = continue/swap screen
    xiv. + = pause game and go to menu ❼ continue playing, retry game or quit and return to exercise menu
    xv. Home button = gives option to go to Wii Menu
    xvi. Arrows = let you navigate through buttons instead of using wii remote as a cursor

| WII U FIT | | | | | | | |
|---|---|---|---|---|---|---|---|
| **Mobility activity** | **Game** | **Game length** | **Description** | **Movement/ Feedback** | **Progress/ Motivation** | **Issues/ Additional demands** | **Rehabilitation modifications** |
| **Preparation for standing up** | WiiFit balance/ Core luge | Game finishes when course complete (running timer) | The player is required to sit on the balance board and shift weight in all directions to maneuver a sled through a course | All directions in sitting/ speed and direction of sled movement, time through course | Nil/ leaderboard, completing course in a faster time | Requires fast AP movement to initially move the sled, requires sitting balance | Place balance board on plinth with thigh support and feet flat on the ground. Reach forward towards a target at the beginning to initiate sled movement. Use dot representing COM rather than person to guide performance |
| **Stepping in Standing** | WiiFit balance/ Hose down | 120secs | The player is required to stand with one leg on the balance board and shoot water at oncoming targets by leaning forward to increase weight through the front leg. In order to 'refill' hose with water, weight must be taken off the front leg (eg. Leaning back or stepping off) Meanwhile, using the game pad to aim the hose at the targets. | AP direction in step stance/ strength of water stream increases with increased weight through leg on balance board, allows errors, score of number of hit targets | None/ leaderboard, increasing score of targets hit | Fast paced, must coordinate UL/LL simultaneously | Set small number of targets to hit during the allocated time, while encouraging full strength of hose (maximum weight bearing through front leg) |
| | WiiFit | 160secs | The player is required | Stepping on spot/ | Nil/ | Mocking, | To decrease challenge, |

| | | | | | | | |
|---|---|---|---|---|---|---|---|
| **Stepping in Standing** | balance/ Dessert course | | to take steps on the balance board to move waiter to pick up a dessert from a chef and deliver the dessert to customers, while holding the game pad level to balance the dessert on the tray | waiter moves proportional to speed of stepping, dessert falls off tray if game pad not held horizontal, allows for error | leaderboard, points (increase number of desserts delivered) | potential risk of falls/ requires fast step frequency in order to move at adequate speed, must coordinate UL/LL simultaneously | allow player to take slower steps, and aim to balance board (record number of dropped desserts in allocated time) |
| | WiiFit balance/ Obstacle course | 80secs | The player is required to step on the balance board to move forward through a course and avoid obstacles in passing | Stepping on the spot/ character moving forward relative to player stepping speed, allows for error, if hit by an obstacle character gets pushed off | Progress to 'Ultimate obstacle course" (see below)/ leaderboard, increasing distance walked in time | Requires fast stepping to avoid obstacles when passing them | Focus on stepping and speed of stepping rather than trying to avoid obstacles |
| | WiiFit balance/ Ultimate obstacle course | 80secs | The player is required to step on the balance board to move forward, turn body and feet to change directions to avoid obstacles and follow course | Stepping on the spot/ character moving forward relative to player stepping speed, allows for error, if hit by an obstacle character gets pushed off | Nil/ leaderboard, increasing distance walked in time | Falls risk with turning/ requires fast stepping to avoid obstacles when passing them | Focus on stepping and speed of stepping rather than trying to avoid obstacles |
| | WiiFit balance/ Scuba search | Game ends when oxygen tank runs out | The player is required to stand on balance board and step *or* squat to swim and simultaneously turn body (anywhere up to 360°) in order to collect fish | Stepping, squatting on the spot/ movement of scuba diver, total score of fish collected at end | Nil/ number of fish collected in time | Falls risk with turning 360°/ need good balance, must change from looking at screen to game board when facing | |

| | | | | | | | |
|---|---|---|---|---|---|---|---|
| | | | | | | backwards | |
| Standing | WiiFit balance/ Bird's-eye Bulls' eye | ~60secs | The player is required to stand on the balance board and flap arms while shifting weight in all directions in order to make the bird fly to and land on targets | Flapping arms while standing on spot and weight shift in all directions/ height of bird, direction of movement | Nil/ leaderboard, increasing number of targets hit, decreasing time to target | Requires vigorous flapping with arms (additional aerobic demand), requires good shoulder ROM and nil shoulder pain | Aim at decreasing time to first target rather than trying to reach as many targets as possible |
| | WiiFit balance/ Snowball fight | 90secs | The player is required to stand on balance board and laterally shift weight to avoid getting hit by snowballs and to position character to throw snowballs at targets by aiming with the Wii remote | ML direction, aim with upperlimb/ allos for errors, character moves left or right from behind the screen, snowball hits target, strength (distance of throw) decreases if you get hit | Nil/ leaderboard, increase number of targets hit in allotted time | Aiming with UL requires finer movements and coordination between UL and LL, requires quick weight shift so as to avoid getting hit by snowballs | Set a small target number |
| | WiiFit balance/Tilt city | ~90secs | The player is required to tilt Wii remote and laterally shift weight while standing on the balance board in order to place balls into buckets according to colour | ML direction (LL), tilting of remote (UL)/ allows for error, tilt of upper bar and lower bars, balls into buckets | Nil/ leaderboard, score of number of balls into buckets | Requires UL/LL coordination, increased cognitive demand with choosing the correct bucket to place balls into | Focus on the number of balls that the player is getting into the buckets |

## *XBOX KINECT*

### Getting started with Xbox Kinect

a. Place sensor at hip height, and ensure you have sufficient play space to play Xbox Kinect.

b. You need the Xbox remote to get started to initially navigate along the Xbox menu. Hold down the silver “X” button to turn the remote on. Use the “A”, “B”, “Y” and “X” buttons and arrows as instructed on the screen.

c. Turn Xbox and TV on without disc in the console.

### Creating an avatar in Xbox Kinect

a. As for the Wii you can create a personal profile for your participant, and this will enable them to work on beating their best score and they may enjoy seeing their own personal avatar on the screen.

b. Use the remote to navigate to the Sign In menu. In here you can select a profile you have already created or you can create a new profile by naming a new profile, using an avatar as a base for your new profile and editing the features of the avatar to match your participant (if you want). Alternatively create a young and older male and female avatar and use these for all your participants.

### Playing a game with Xbox Kinect

a. Place the chosen software disc in console and it will go directly to that game. Note that you can create a new profile once you are in the game, you will need to sign out the current profile and then it will let you create a new profile.

b. Once the software is open, you no longer need the remote and you can use your hand to navigate through the software to the game you want to play.

c. If you need to pause a game at any time, hold your left arm out 45° and hold it there until the game pauses.

d. The software we have investigated so far include: Kinect Adventures, Kinect Sports, Fruit Ninja (located in games library), Dance central, and your shape evolved 2012. Please note

   a lot of the Kinect games are very fast or require 2 arms to be used or the person to jump, so they are not appropriate for our participants. A lot are also not particularly specific to mobility limitations and don't provide feedback regarding good movement patterns. However, the games are very engaging and fun to play, and may be more useful for our higher-level participants to push their mobility limitations and to increase their overall physical activity.

| KINECT ADVENTURES | | | | | | | |
|---|---|---|---|---|---|---|---|
| **Mobility activity** | **Game** | **Game length** | **Description** | **Movement/ Feedback** | **Progress/ Motivation** | **Issues/ Additional demands** | **Modifications** |
| **Stepping and reaching in standing** | **Freeplay/ 20000 leaks (3 games leaks ahoy, go with the flow, ship shape)** | ≥ 2mins | The player is required to repair leaks infront, side and on floor by reaching and stepping and holding that position on leaks to plug them. | Step and reach in different directions/ score at end, bonus points finishing early | Ingame 3 waves, ↑duration and number of leaks at once, 3 games increase difficulty /, allows error | Timing, accuracy of hand and foot placement | Could just plug wall ones with hand and not step. |
| **Stepping in standing and jumping** | **Freeplay/ River rush (3 games curvy creek bouncing brook cozy cavern)** | ≥ 2mins | The player is required to make their way in a raft down a river by jumping to start, and stepping side to side to miss obstacles and to gain pins (which give points), and jumping to get high pins. | Jumping, side to side stepping/ score at end | No, different river courses for 3 games/ allows errors | Need to jump to start game, fast paced, timing to get pins | Pushing up on toes is perceived as jump by sensor, particularly fast up on toes. |
| | **Freeplay/ Reflex ridge (3 games mover, collector, cruiser)** | ≥ 2mins | The player is on a railway track. They must collect pins (A) while avoiding obstacles by side stepping, ducking or jumping. | Jumping, side to side stepping, ducking/ score at end | No, different railway tracks for 3 games/ allows errors | Need to jump and duck to get over some obstacles, need to pull through with 2 hands to start the game. | Pushing up on toes is perceived as jump by sensor, particularly fast up on toes. |

| | | | | | | | |
|---|---|---|---|---|---|---|---|
| **Stepping in standing** | Freeplay/ Rally ball (3 games sure shot, treasure chest, peekaboo) | ≥ 2mins | The player is required to make their way in a raft down a river by jumping to start, and stepping side to side to miss obstacles and to gain pins (which give points), and jumping to get high pins. | Jumping, side to side stepping/ score at end | No, different river courses for 3 games/ allows errors | Need to jump to start game, fast paced, timing to get pins | Pushing up on toes is perceived as jump by sensor, particularly fast up on toes. |
| | Freeplay/ Space pop (3 games Blast off, Halo hopper, Solar streaks) | ≥ 2mins | The player is in space in ship. They must pop all the bubbles by flapping both arms to make them go up in the air, and stepping side to side and forward and backward to get all the bubbles. | Stepping forward, backward and side to side while flapping arms. | No, 3 different games/ allows errors | Need both arms to flap to float up in the air | Nil |
| **KINECT YOUR SHAPE FITNESS EVOLVE 2012** | | | | | | | |
| **Shifting weight in standing** | Warm-up/ juggle it | | Keep the soccer ball in the air with no hands by juggling it between your head, knees and foot | Heading, kneeing or kicking virtual soccer ball/ | Unlock harder levels/ beating previous score | | Nil |
| | Warm-up/ kick it | 2 mins | Kick the ball through goals | | | | |
| | Warm-up/ hurricane | 2 mins | Stand and spin your arms together to create a hurricane of balls, 30secs then swap direction of arm spin | | | | |
| | Warm-up/ Pump it | | Shoulder abduction single arm or together, small range or large, Get points for the number | | | | |

| | | | | | | | |
|---|---|---|---|---|---|---|---|
| | | | of balls blown up and popped. | | | | |
| | Warm-up/ Hula la | | | | | | |
| **Maintaining a standing position** | Stack em up | 2mins | Hold the virtual board in your hands, catch the falling blocks, stack them on the board, drop into the container on either side. To grow your board stand on 1 leg, hold more blocks on the board to get more points | Standing with arms infront, move arms to empty board, single leg stance/ allows errors | Unlock harder levels/ beating previous score | Focus is on calories/ Dual task cognitive + UL task | |
| **Stepping in standing** | Stomp it | 2mins | Step forward or to either side | Stepping forward and to sides/KOR colour lights up when get correct timing, score at end | Unlock harder levels/ beating previous score | Focus is on calories very fast paced and cognitively challenging, timing element/ Dual task cognitive | Modify by selecting just 1 colour only to step on e.g. step on purple if you want to practice stepping with left foot. |
| **Reaching in standing/ changing direction** | Wall breaker | 2mins | Punch arms across body at 45 degrees to break cubes on the screen | Rotating on the spot and punching side to side/ KOR block disappears when hit it, total score at end, allows error, tell you can do better | Unlock harder levels/ beating previous score | If you don't punch hard enough it does not register, focus is on calories/ Dual task motor | Nil |
| **Physical activity/ Walking** | Run the world | ?? | Follow course through New York city. | Walking on the spot/ KOR gives time for distance walked | Unlock harder levels/ beating previous score | Speed not an issue, can stop and rest | Nil |

| | | | | | | |
|---|---|---|---|---|---|---|
| | **KINECT SPORTS** | | | | | |
| **Reaching while standing, stepping and Standing** | Mini-game soccer (super saver) | The participant is required to reach and or step and reach side to side to stop the soccer ball | Reaching side to side and stepping side to side/ KOR gives score, allows error | Progress to full soccer game/ beating previous score | Reasonably fast paced/ timing and dual task UL | |
| | **KINECT DOWNLOADED GAMES** | | | | | |
| **Maintaining a standing position** | Fruit Ninja | The participant must maintain a standing position while cutting the fruit on the screen | UL movements in standing position | Different games e.g. bomb included which you can't hit/ previous score | Have to slice fruit fast enough to register | Use zen mode to begin as no bomb and allows you to miss fruit. |

## *HUMAC 2013 (v.150)*

Weight capacity = 150kg

### Getting started with HUMAC

a. Ensure balance board is plugged into computer.

b. Open the HUMAC software program on the desktop icon.

c. From the menu bar file option, select Preferences.

d. Under the General tab, ensure the interface section has “balance board” checked.

e. Under the Reporting tab, ensure metric units are selected.

f. Click the **OK** button to read the zero weight (ensure nothing is on the balance board). The HUMAC will return to the Main Menu.

### Adding a new participant into the HUMAC

a. From the main screen, click the patient button.

b. Select New patient button and add participant details. You have to enter DOB, height and weight.

c. After adding the participant information click OK to save new patient information and return to the main HUMAC screen.

d. The new patient name will now display in the top Title bar and all the buttons on the button bar to commence testing and exercise will be active.

## Commence exercising or testing

a. If you have already created your participant profile, select that patient using the patient button. Check that the participant you are working with is listed in the top title bar.
b. From the button bar select either "test" if you want to conduct an initial test or progress test, "exercise" to commence exercises with your participant or "dashboard" if you want to play any of the games e.g. pong (note: in dashboard mode the games are not played under a particular participant)

## Other features

a. The manual explains how to create test protocols, conduct reports to compare the same test protocol for the same participant over time, how to get usage reports (this details all the tests and exercises completed for each participant so will be good for process evaluation), how to back up data (this will be important).

## Participant setup on the board

a. Get the participant to stand on the board and when participant setup screen appears before each exercise, record the foot angle, position of medial malleolus and middle of heel using the letters and numbers displayed on the board (see page 56 manual)

b. Next set anatomical zero for participant. Ask the participant to stand in as neutral position and select OK. Whatever position they are in when you select OK this will be taken as neutral position.

| HUMAC | | | | | | |
|---|---|---|---|---|---|---|
| **Mobility activity** | **Game** | **Game Length** | **Description** | **Movement / feedback** | **Progress/ Motivation** | **Issues/ Modifications** |
| **Maintaining a standing position** | **Clinical Test of Sensory Organization (CTSIB) eyes open, firm surface** | Select: 15, 30, 45, or 60secs, OR 1, 1:30, 2, 5, or 10 minutes | Maintain EWB position to enable the ball to stay central on the target | Maintain EWB/ KOP keep ball on target or changes colour, graph KOR | Increase time, add foam or balance board or eyes closed; progress onto different game/ graph 2 games on different days to show improvement (test mode only) otherwise print graphs for 2 different days | |
| | **Weight Bearing** | Sets: 1,2,3,4,5,10, 15,20, 25<br>Rest b/n sets: 15, 30,45,60secs<br>Duration: 10, 15, 30, 45secs, OR 1, 1:30, 2, 5, or 10mins | The participant needs to maintain COP in neutral position in either AP or ML direction | Maintain COP in neutral position in either AP or ML direction/ KOP graphical display with written percentages of WB, KOR: report graph error | Duration and sets / KOP within game, KOR: report graph error | |
| | **Weight Bearing XY** | Sets: 1,2,3,4,5,10, 15,20, 25<br>Rest b/n sets: 15, 30,45,60secs<br>Duration: 10, 15, 30, 45secs, OR 1, 1:30, 2, 5, or 10mins | The participant needs to maintain COP in neutral position in both AP or ML direction | Maintain COP in neutral position in both AP or ML direction/ KOP graphical display with written percentages of WB, KOR: report graph error | Duration and sets / KOP within game, KOR: report graph error | |

| Mobility activity | Game | Game Length | Description | Movement / feedback | Progress/ Motivation | Issues/ Modifications |
|---|---|---|---|---|---|---|
| Shifting weight in standing | **Centre of Pressure** | Sets: 1,2,3,4,5,10, 15,20, 25<br>Rest b/n sets: 15, 30,45,60secs<br>Duration: 10, 15, 30, 45secs, OR 1, 1:30, 2, 5, or 10mins | The goal is for the participant to keep the magenta cursor in the middle of the bulls eye by maintaining their COP in the neutral position | Maintain COP in neutral position/ KOP can see on grid where magenta cursor, therefore COP is located, KOR: graph with stability score and percentage of time spent in each quadrant and ring | Increase duration and sets, reduce rest time between sets/ KOP within game, KOR graph, compare between sessions. | Most useful for person with increased postural sway, may use as measurement to track progress. |
| | **Stability** | 1-25 sets, rest 0-2minutes between sets; hold at each number on the clock for 1-10 secs | Shift your weight clockwise around face of a clock starting at 12. Keep the target green at each point by maintaining your weight at each point (target yellow when not correct) | Full circle around your neutral position, how much movement depends on level set/ KOP: target is green when COP is in correct position, yellow when not; KOR: % of time on target for each position, can graph | Levels 2-8 represent how far you have to move your weight in the different directions/ graph 2 games on different days to show improvement (test mode only) otherwise print graphs for 2 different days | Shorter time e.g. 5secs have to move quite quickly between targets, 10sec probably good to begin. |
| | **Mobility** | 1,2,3,4,5,10,15,20, 25 sets, rest 15,30, 45, 60 secs between sets; time for each set 10, 15, 30, 45secs, 1,1:30, 2, 5, 10mins | Shift your weight clockwise by holding your COP on the moving target that circles around your neutral position. | Full circle around your neutral position, how much movement depends on level set, how fast depends on time set/ KOP: target is green when COP is in correct position, yellow when not; | Levels 2-8 represent how far you have to move your weight in the different directions/ graph 2 games on different days to show improvement (test mode only) otherwise print graphs for 2 different days | Shorter time e.g. 5secs have to move quite quickly between targets, 30sec probably good to begin. |
| | | | | KOR: % of time on target, can graph. | | |

| | Game | Game Length | Description | Movement / feedback | Progress/ Motivation | Issues/ Modifications |
|---|---|---|---|---|---|---|
| **Shifting weight in standing** | **Stability envelope** | 1,2,3,4,5,10,15,20, 25 sets, rest 15,30, 45, 60 secs between sets | The arrow shows the direction the participant should lean ( 0°, 45°, 90°, 135°, 180° 225°, 270°, 315°). The magenta cursor shows the participant COP. Therapist clicks the mouse on cursor when the participant reaches their furthest distance at each angle. | As far as participant can move in the direction of 0°, 45°, 90°, 135°, 180° 225°, 270°, 315° from neutral position. Self-paced. | Nil progress, measurement/ graphs total area of movement, table of area for each direction moved. | May be more useful for measurement of improvement in maximal excursion of COP in all directions. |
| | **Weight Shift** | Sets: 1,2,3,4,5,10, 15,20, 25<br>Rest b/n sets: 15, 30,45,60secs<br>Reps: 5, 10, 15, 25, 30, 40, 50<br>Self-paced | The goal is for the participant to move through the green marker in the correct direction while staying inside the magenta boundaries | ML direction (0° rotation)/ KOP cursor stays magenta if within set boundaries, red if out of boundaries, magenta cursor moves towards green line as you move COP, KOR graph trace of COP movements, Score number of reps within boundaries and time taken to complete set reps. | Increase sets and/or repetitions, decrease rest between sets, increase distance have to move, decrease boundaries have to work between/ KOP within game, KOR graph, compare between sessions. | Can set different distance targets for each direction, larger set boundaries easier for participant |

| | Game | Game Length | Description | Movement / | Progress/ Motivation | Issues/ Modifications |
|---|---|---|---|---|---|---|

| | | | | | | |
|---|---|---|---|---|---|---|
| | | | | feedback | | |
| Shifting weight in standing | **Limits of Stability** | Sets: 1,2,3,4,5,10, 15,20, 25<br>Rest b/n sets: 15, 30,45,60secs<br>Hold time on each target: 1-60secs<br>Total duration: self paced | The goal is for the participant to move their magenta cursor to the flashing yellow and black target by shifting their COP. The targets are selected in random order and the participant must return to the centre target between each outer target. All 8 outer targets must be completed to finish the set. | Random movement of COP in 8 directions (0°, 45°, 90°, 135°, 180° 225°, 270°, 315° around neutral position) distance to move dependent on level set/ KOP target turns green when cursor is held on the correct target, turns yellow if move off target before the hold time is up, KOR graph of COP trace and table showing % score compared to normal for path taken to get to target and average time taken to get to target | Increase level 2-8 (distance to target), reps and hold time, decrease rest between sets/ KOP within game, KOR graph, compare between sessions. | Therapist can skip a target if participant is having difficulty getting that target. |
| | **Targets** | As for limits of stability | As for limits of stability but tailored for the participant. You get the participant to move AP and ML as far as they can go and set these as their limits. | Movement in different directions that the targets have been placed by the therapist, the order the targets are added to the screen are the order they are selected/ feedback | Increase distance therapist sets the targets at, reps and hold time, decrease rest between sets/ KOP within game, KOR graph, compare between sessions. | The program enables you to set limits of movement first so that you can target a particular area. This may be more suitable than limits of stability if person has significantly different distances they |

| | | | | | | |
|---|---|---|---|---|---|---|
| | | | You can then add targets around their neutral position within | as for Limits of stability | | can move their COP in the different directions. |

| | Game | Game Length | Description | Movement / feedback | Progress/ Motivation | Issues/ Modifications |
|---|---|---|---|---|---|---|
| | | | their limits. | | | |
| **Shifting weight in standing** | **Random Motion** | Sets: 1,2,3,4,5,10, 15,20, 25<br>Rest b/n sets: 15, 30,45,60secs<br>Duration: 10, 15, 30, 45secs, OR 1, 1:30, 2, 5, or 10mins | The goal is for the participant to keep their cursor on the randomly moving target (speed of target is set between 1[slow] to 5 [fast]) | Movement of COP in different random directions, change of direction/ KOP target is green when your magenta cursor is on the target, yellow when your cursor is not on the target. KOR: graph of trajectory of COP, % time on target. | Increase speed, sets, duration of game, decrease rest between sets, set greater limits of movement/ KOP within game, KOR graph, compare between sessions. | Set limits of movement first, can target a particular area when setting limits. |
| | **Roadway** | Sets: 1,2,3,4,5,10, 15,20, 25<br>Rest b/n sets: 15, 30,45,60secs<br>Reps: 5, 10, 15, 25, 30, 40, 50 | The goal is for the participant to keep the round cursor between the roadway boundaries by moving their COP | Can be set ML or AP/ KOP green cursor indicates within boundaries, red cursor out of boundaries, changing score dial % of time within boundaries, KOR graph displays COP vs. time overlaid on roadway and % of time on target for raising (to right or anteriorly-concentric) or lowering (to left or posteriorly-eccentrically) | Can change the profile of the roadway (steeper rise or fall meaning you have to change direction quickly), level 1 (easy) to 5 (hard), [accuracy set to 0], can increase sets, reps and decrease rest period/ KOP within game, KOR table and graph, compare between sessions. | This is quite challenging even level 1 and super slow profile as you need to work out and remember which direction you need to move. Easier/more intuitive when working in the AP direction. Increased cognitive demands/less intuitive when working in the ML direction of movement. May also use force mode to practice generating force through affected lower limb (through placing unaffected limb on block next to board). |

| Mobility activity | Game | Game Length | Description | Movement / feedback | Progress/ Motivation | Issues/ Modifications |
|---|---|---|---|---|---|---|
| Standing up from a chair | **Force vs. Time** | Sets: 1,2,3,4,5,10, 15,20, 25<br>Rest b/n sets: 15, 30,45,60secs<br>Reps: 5, 10, 15, 25, 30, 40, 50<br>Self-paced | Ask the participant to stand up as quickly as they can at the beginning of each repetition, stay standing for repetition, and then sit in rest period between repetitions. | Standing up and sitting down/ KOP force vs. time graph is displayed, slope of curve shows speed of extensor force generation; KOR: table average peak force % BW and average time to peak force. | Can modify duration of repetition (i.e. time to stand up, hold time 1-60secs), duration of rest between repetitions (i.e. time to sit down and prepare for next STS, relax time 1-300 secs), ?force threshold, increase number of sets & repetitions, decrease rest time between sets/ KOP within game, KOR table and graph, compare between sessions. | ?? unclear proper use of this exercise. STS seems to work well if wanting to work on someone's speed of force generation. Can target weaker side by putting other foot on block next to board. |
| Sitting balance | **Scale** | Sets: 1,2,3,4,5,10, 15,20, 25<br>Rest b/n sets: 15, 30,45,60secs<br>Reps: 5, 10, 15, 25, 30, 40, 50<br>Self-paced | Can use this set up for anything you would use scales for feedback for 1 foot e.g. sitting balance pushing through heel, step stance shifting weight forward | Apply force through leg on board to increase marker on the scales/KOP analogue scale shows weight through board, keeps line for maximal weight for each repetition; KOR: table average peak force % BW and average time to peak force. | Can modify duration of repetition (i.e. time to load the leg 1-60secs), duration of rest between repetitions (i.e. time to load other leg, relax time 1-300 secs), ?force threshold, increase number of sets & repetitions, decrease rest time between sets/ KOP within game, KOR table and graph, compare between sessions. | ?? unclear proper use of this exercise. |

# HUMAC GAMES

| Mobility activity | Game | Game Length | Description | Movement / feedback | Progress/ Motivation | Issues/ Modifications |
|---|---|---|---|---|---|---|
| Shifting weight in standing | **PONG** | Choose number of balls per set (5,10,15,25,30 40, 50, unlimited), number of sets (1,2,3,4,5,10,15,20, 25) and rest between sets (10, 15, 30, 45,60secs). Each ball lasts until you or computer miss it. | Hit the ball back with your paddle to your opponent (computer) by shifting your COP | Choose COP shifts ML or AP (set participants ROM)/ KOP paddle moves up and down as you shift your weight, KOR whether you miss the ball. | Compete against computer, however computer didn't lose when I played so may lose motivation/KOP and KOR within game, keeps score between computer and participant and states at end of sets who won. | Timing element of task. Set range of motion not to participant's limits otherwise hard to move paddle full length. Set ball speed & acceleration low (1-3), paddle (8)and ball size (3) high N.B. the ball speed gets faster as it is hit between the paddle, but therapist can slow down again. Can also set computer skill (0 beginner, 100 expert, at 50% computer still won each point). More useful when training AP movements. |
| | **BREAKOUT** | As for Pong | Move the paddle to knock the blocks out of the wall by shifting COP. | Choose COP shifts ML or AP (set participants ROM)/ KOP paddle moves up and down as you shift your weight, KOR whether you miss the ball. | Can limit the number of balls . | Same issues as Pong. Better option when training ML movements. |

| | | | | | | |
|---|---|---|---|---|---|---|
| | **BALANCE** | Dependent on how long it takes to get the ball on target | Shift COP R/L and A/P to tilt the board to move the ball through a maze into the | Move COP R/L and A/P (set participant ROM) / KOP board on screen tilts in the direction you move, | Level 1-15/ Compete to beat each level best time | If slow movement then ball can get stuck on edges. Table tilt in WiiFit has better sensitivity. |

| | | | | | | |
|---|---|---|---|---|---|---|
| | | | target hole. | KOR get ball on target and game finishes, score is the time it takes to complete | | |
| **Shifting weight in standing** | **SKI** | 1-2min per level (depends how long to ski down the hill) | Ski race where you lean forward on the board to ski down the hill and shift COP to R and L to turn | Keep COP anterior and move R and L/ KOP see the skier turn as you shift weight R or L, score is the time it takes to complete | Level 1-12/ Compete to beat each level best time | Game doesn't indicate if going in wrong direction. |
| | **SNOWBOARD** | 1-2min per level (depends how long to board down the hill) | Same race as ski, but on a snow board. Lean L or R (board turned side on) on the board to ski down the hill and shift COP to AP to turn | Keep COP on front leg and move AP/ KOP see the boarder turn as you shift weight A or P, score is the time it takes to complete | Level 1-12/ Compete to beat each level best time | Game doesn't indicate if going in wrong direction. Good for loading affected leg as front leg (if wrong leg is used player won't progress downhill). |
| | **Game** | **Game Length** | **Description** | **Movement / feedback** | **Progress/ Motivation** | **Issues/ Modifications** |
| | **LUGE** | 1-2min per level (depends how long to board down the hill) | Same race as ski, but on a luge. Lean forward on the board to move down the hill and shift COP to R and L to turn it | Keep COP anterior and move R and L/ KOP see the luge turn as you shift weight R or L, score is the time it takes to complete | Level 1-12/ Compete to beat each level best time | This game appears to still be under construction, can only play in demo mode (can choose upright or reclined, just what the person in the luge looks ike) |
| **Shifting weight in standing** | **FLIGHT** | > 1min each level | Shift COP Anterior to make plane descend, Posterior to | AP and R/L / KOP see plane turn as shift weight, score is time taken to hit 3 targets. | Level 1-12/ Compete to beat each level best time | Can be difficult to get target if shifts weight quickly. |

| | | | | | |
|---|---|---|---|---|---|
| | | make ascend, L and R to turn. Steer the plan to hit targets. | | | |
| **ANIMAL ADVENTURE (not available new software)** | | Shift weight to place the ball on the appropriate target | Pt's own pace | No feedback, gives score | Shifting weight towards all directions and maintain position for fixed seconds and tests pt's cognitively visual and audibly |
| **PACMAN (not available new software)** | | Eat the pallets and don't get captured by the ghosts | Quick reactions | No feedback, gives score | Shifting weight L)↔R), F)↔B) |
| **Game** | **Game Length** | **Description** | **Movement / feedback** | **Progress/ Motivation** | **Issues/ Modifications** |
| **EGG DROP (not available new software)** | | Catch the egg with the spoon by lining up the shadows on the floor | Quick reactions, fast pace | No feedback, gives score | Shifting weight towards all directions |
| **SPACE INVADERS (not available new software)** | | Avoid the missiles by shifting your weight | Quick reactions, (timer: 1,5 minutes, infinity and custom) | No feedback | Shifting weight L)↔R) |

# *FYSIOGAMING 2015 (v2.1)*

## Set-up

- Prior to launching the software ensure the sentinel key (blue USB stick) is plugged into the computer and the Kinect sensor is connected.
- Play space: ideally Fysiogaming requires 1.55m wide each side of the Kinect sensor, and 3.2m distance from the Kinect sensor, with the play area starting 1.3m from the Kinect sensor.
- Launch Fysiogaming software from the desktop icon. The home page has tabs across the top “Patient”, “Module”, “Exercise”, “Play” and “Results”. The software launches in the patient tab. The most recent player will be shown on the screen.
- Kinect sensor location: The Kinect sensor height should be correctly defined. Depending on the height of the patient you may wish to increase or decrease the sensor height. In the patient tab go to **“Settings”**. Measure the distance from the ground to the base (foot) of the Kinect. Type the number (height in cm) and click save. Click on **“Save”** to accept the changes.

## Adding a new participant

- Select “New patient” button in Patient tab.
- Enter participant data e.g. first name and initial for the surname (remember de-identified participant data for final storage of electronic and paper copies- you can go in and modify the participants data to be their participant ID once they have finished using the system.
- Note: ***** means a required field.
- Enable capture patient photo unless the participant specifically says not and a picture will be taken of the participant from the Kinect sensor.
- Calibrate: select “every time” for the first session and then go in and change to “use previous calibration”.
- Breakdown: this allows you to customize rest periods between sets of exercises (exercises) and between different exercises (series).
- Save changes once you have completed this screen and that participant is now added to the system.

## Patient Tab

From the patient tab you can select your participant to commence exercising. If it is the participant you just entered then you are ready to go.

- Other buttons on the patient tab include:
    - Patient data: displays basic patient’s data and enables you to edit details about the selected participant.

- **Statistics** – click here to view training session history and statistics of the selected patient.
- **Details** – click to see the details of the last training session (see Figure 1)

## Starting to exercise

- On the right hand side of the patient tab there are 4 Options to starting a training session:
  a) **New program** – click here to create a new training program.
  b) **Reapply last** – click here to repeat the last training program.
  c) **Increase level** – click here to repeat the last training program with the difficulty level increased by one.
  d) **Adjust program** – click here to adjust the last training program.
- To create a new program you will be taken to the Exercise (or Module) Tab where you can navigate through different types of exercises (see table below) and select reps and sets of each exercise to create a total program. As you select each exercise the total time of exercise for the whole program is displayed at the top right hand corner.

## Difficulty level

Fysiogaming offers 30 difficulty levels which are divided into three groups:

- 1-10: Easy
  *Performance*: Exercise can be performed with low precision.
  *Speed*: The speed is very low.
  *Games*: A required reaction time is high. Playing and gaining points is easy.
- 11-20: Medium
  *Performance*: The application requires moderate precision in performing exercises.
  *Speed*: The speed is on a medium level.
  *Games*: Games are more demanding in terms of reflexes and reaction time than on levels 1-10.
- 21-30: Difficult
  *Performance*: Exercises require high accuracy.
  *Speed*: The speed is on a high level.
  *Games*: Required reaction time is small. The games become more difficult and more challenging.

## Assessment Centre

- You can use this section to get a repeat measure on participants' performance. You can assess:
  - Sit to stand: time for 1 repetition, time for 5 repetitions, number of repetitions in 30secs
  - Trunk balance Ax seated movement:

- Trunk balance Ax standing movement:

## Reviewing and reporting participant data

- Within the statistics button on the patient tab you can review the session history.
    - Reports: You can save/print graphs of the participants movements during the games
    - Details: You can save/print exercises/games completed including program, reps, duration, score and difficulty level.
    - Re-apply: to start program again.

Adjust program: click here to adjust the last training program.

| FYSIOGAMING | | | | | |
|---|---|---|---|---|---|
| **Mobility activity** | **Game** | **Description** | **Movement / feedback** | **Progress/ Motivation** | **Issues/ Modifications** |
| **Stepping in standing** | **Walking sideways** | The player steps to L and R to move the boat to catch the falling gems and to miss the barrels. | Side stepping to right and left/ KOR score at end, allows error | Game can be set from level 1 (easy) to level 30 (hard) which reflects accuracy/ score and level of game, medals and photos at end of session | Need sufficient room to step side to side/ timing and cognitive demands to get gems and miss barrels. |
| | **Hip abduction** | The player moves their leg to the L and/or R to move the boat to catch the falling gems and miss the barrels. | Hip abduction to right and left/ KOR score at end, allows error. | Game can be customized to be set from level 1 to level 30 (increasing difficulty reflects accuracy/speed), ROM of legs (50% or 100%), limbs involved (left/right or both) and movement type (controlled VS. dynamic). Medals and photos at end of session | Difficult for therapist to provide standby/physical assistance as may get in way of movement. May use for eccentric training of hip abductors in stance limb. |
| | **Side strides** | The player must lunge to the L and R to lay down pillars to build a pergola. | Side lunges to the right and/or left/ KOR score at end, allows error | Game can be customized to be set from level 1 to level 30 (increasing difficulty reflects accuracy/speed ), ROM of legs (50% or 100%), limbs involved (left/right or both) and movement type (controlled VS. dynamic). Medals and photos at end of session | Need sufficient room to step side to side. Difficult for therapist to provide standby/physical assistance as may get in way of movement. |
| | **Lunges (forward)** | The player must lunge forward alternating between | Forward lunges to the right and/or left. KOR score at end, allows error | Game can be customized to be set from level 1 to level 30 (increasing difficulty reflects | Need sufficient room to step forward. |

| | | | | | |
|---|---|---|---|---|---|
| | | the L and R legs to row the boat down the stream | | accuracy/speed), ROM of legs (50% or 100%), limbs involved (left/right or both) and movement type (controlled VS. dynamic). Medals and photos at end of session | |
| | **Knee and hip flexion** | The player must flex at the hip and knee to lay down pillars to build a coliseum. | Hip and knee flexion on the right and/or left. KOR score at end. Allows error. | Game can be customized to be set from level 1 to level 30 (increasing difficulty reflects accuracy/speed), ROM of legs (50% or 100%), limbs involved (left/right or both) and movement type (controlled VS. dynamic). Medals and photos at end of session | May use block to make into a step touch exercise. Increase block height/use of polystyrene cup to increase difficulty. |
| | **Knee flexion (hip neutral)** | The player must flex at the knee with the hip in neutral position to lay down pillars to build a pergola. | Knee flexion with the hip in neutral position. R and/or L LLs may be used. KOR score at end. Allows error. | Game can be customized to be set from level 1 to level 30 (increasing difficulty reflects accuracy/speed), ROM of legs (50% or 100%), limbs involved (left/right or both) and movement type (controlled VS. dynamic). Medals and photos at end of session | Sensor may not always pick up movements posteriorly. |
| | **Dynamic balance forward and backward/ Dynamic balance side to side/ Dynamic balance forward and to the sides/ Dynamic balance-X/ Dynamic balance-cross/** | A 9 cell grid is displayed on the screen. The player must step in the direction of the flashing cell on the grid (this direction will depend on which game you select) | Step in ML/ AP/ diagonal direction/ KOR score at end, allows error | Game can be set from level 1 (easy) to level 30 (hard) which reflects accuracy/ score and level of game, medals and photos at end of session | Need sufficient room to step in different directions |

| | | | | | |
|---|---|---|---|---|---|
| | **Dynamic balance-random** | | | | |
| | **Walk in place** | A walking track is displayed on the screen. The player is required to walk on the spot and step side to side to collect gems and miss holes in the ground | Walking on the spot, step side to side/ KOR score at end, allows error | Game can be set from level 1 (easy) to level 30 (hard) which reflects accuracy/ score and level of game, medals and photos at end of session | Need sufficient room to step in different directions |
| **Shifting weight in standing** | **Leaning trunk to the sides/ Leaning trunk forward and backward/ Leaning trunk forward, backward and to the sides/ leaning trunk to diagonal directions/ Leaning trunk to all sides** | A 9 cell grid is displayed on the screen. The player must move their COP in the direction of the flashing cell on the grid (this direction will depend on which game you select) | ML/ AP/ diagonal displacement of COP/ KOR score at end, allows error | Game can be set from level 1 (easy) to level 30 (hard) which reflects accuracy/ score and level of game, medals and photos at end of session | Game movement is more trunk LF, to promote movement at the hips , get the person to reach with their arm in the direction of the flashing cell. |
| **Reaching while standing** | **Reaching** | An ocean ground is displayed on the screen. The player is required to reach forward in different directions using R and L hand as instructed | Moving COP and reaching in direction of target/ KOR score at end, allows error | Game can be set from level 1 (easy) to level 30 (hard) which reflects accuracy/ score and level of game, medals and photos at end of session | Can tailor program for single arm use in hemiplegic patients |
| **Standing up from a chair** | **Sit to Stand** | An ocean ground is displayed on the screen. The player must stand up and sit | Standing up and sitting down/ KOR score at end, allows error | Game can be set from level 1 (easy) to level 30 (hard) which reflects accuracy/ score and | The timing of the movement is not in sync |

| | | | | | |
|---|---|---|---|---|---|
| | | down to move the submarine Up (standing up) and down (sitting down) to collect the coins | | level of game, medals and photos at end of session | with what is displayed on the screen. |

## *LusioMATE*

LusioMATE is a wearable gaming device that can be prescribed to as an add-on to physiotherapy practices.

### Getting started:

1. It requires an iPad with Lusio game apps installed, two charged sensors, and few straps to keep sensors in position on the body part.
2. Connect the iPad to the Internet using the User name: __________________________ and Password: _______________________
3. Turn on the LusioMates - the two small white sensors- by moving the small black switch on its side. They will turn red.

### Registering a participant:

1. Open the LusioHub icon. Enter the Email ID ________________ and Password ____________
2. Press 'Register a new client'. Enter Participant ID in user name, the participant's initials in the First name and Last name, their Gender, and any Date of Birth (do not put original). Press 'Register'. Exit the icon by pressing the exit button on the top right-hand corner.

### Setting up a game for the Participant for the first time 'By Workout':

1. Choose a game app that you want your participant to play according to the type of exercise or movement you want to train. There are about 24 games to choose from. Some of the most useful games are Jumpy Rabbit, Basketball, Steelman, Endless racing, Racoon rescue, Flying rocket, Sliding Santa, etc. Please refer to the table at the end of this section for the details on each game and what games to choose to train common rehabilitation exercises.

3. Log in into the game by using the Username: __________________ and Password: _____________
4. Press 'Create' to create a set of exercises for the participant 'By workout'.
5. Choose and click one from a selection of predefined workouts.

6. Follow the prompts and enter the Personal goal, Time and Reps that you want the client to achieve. Also select left or right side, and the placement of sensors by clicking on one of the pictures provided. For e.g. left ankle, left knee ect. The placement of the sensors on the participant should be like the picture selected, therefore do remember the placements.
7. Click on 'Forgiveness' if you think your participant would need some flexibility around the range of motion that the participant can achieve with each rep. For eg. this feature can be used if the participant movement is variable and cannot achieve the same movement with each repetition. The 'Forgiveness' can be set to 20, 40, 60, 80 or 100%. The more the percentage is, the less strict it is in achieving the movement/range of movement during the game. Press 'Done' when finished.
8. Click on Easy, Medium or Hard according to the level you think your participant can perform (Start with Easy if it is the first time and you can change it to medium or hard if needed the next time).
9. Hold the sensors in your palm and move it in a figure of eight pattern for 10-15 sec to calibrate the sensors. Attach the sensor on the body part of the participant using straps like in the picture that you chose in no.6
10. In the 'Position Set up' screen to set up the start and end position of the range of movement that you want to train.
11. Click on Automatic or Manual mode.
12. If you click on Automatic mode, the iPad will automatically pick up the joint range of the movement that you want to train when you ask the participant to do that movement. Ask the participant to do the movement many times until a circle completes on the screen, usually 5 to 8 times.
13. If you are clicking Manual, you have the independence to choose the start and end positions of the range of the movement that you want to use/train. For this, press 'Set start' and ask the participant to move through the range you want and then press 'Set End' when you want to stop or when the patient stops. This will set the start and end range of the movement that you are intending to train.
14. After the circle forms, you need to wait for the red button on the horizontal white line to move freely as the participant moves through the range. Until then keep doing the movement a few more times. Sometimes there can be a small delay.
15. After the red dot starts moving, press Play.

16. This will start the game and when the participant moves through the range that you have set, they play the game that you have chosen. Most games last a minute and counts down from 60 sec. The countdown time can change according to the game. The repetitions come on the screen**. Please record the repetition and time played in the technology recording sheet and keep it safe in the AMOUNT2 folder under each subject ID.** There is a pause button and play button if you need to pause and then play. The game can be exited using the exit button on the top right-hand corner of the screen. You can also press ‘Back’ and go back to recalibrate the sensors. Sometimes, if the program hangs, you might have to trouble shoot by exiting the game and starting over again.

17. The game can be continued unlimited times by either waiting for the game to re-start on its own after certain seconds depending on the game or by pressing the restart button.

18. You can progress by choosing medium or hard in each game if you think your participant requires more challenging games. This can be done by going ‘Back’ to the ‘Prescription’ window and choosing ‘Medium’ or ‘Hard’ levels.

19. You can ‘Create’ as many games as you want for a participant and it gets saved under their ID when you open each game.

### Setting up a game for the Participant for the first time ‘By Joint’:

1. Choose a game app that you want your participant to play according to the type of exercise or movement you want to train. There are about 24 games to choose from. Some of the most useful games are Jumpy Rabbit, Basketball, Steelman, Endless racing, Racoon rescue, Flying rocket, Sliding Santa, etc. Please refer to the table at the end of this section for the details on each game.

2. Log in into the game by using the Username: ________________and Password: ______________

3. Press ‘By Joint’ to ‘Create’ to create a set of exercises for the participant.

4. Follow the prompts and enter the personal goal, time and reps that you want the client to achieve.

5. ‘Select a Joint’ by clicking on one of the joints of the lower limb (hip, knee or ankle joint) of image of the human body provided. This will bring up different movement options on the right-hand side. For eg. If you click on hip joint, it will bring up flexion/ extension and abduction/ adduction movement options for that joint. Click on the movement that you want your participant to train. You can choose left or right side by choosing left or right on the human body image.

6. Click on 'Forgiveness' if you think your participant would need some flexibility around the range of motion that the participant can achieve with each rep. For e.g. this feature can be used if the participant movement is variable and cannot achieve the same movement with each repetition. The 'Forgiveness' can be set to 20, 40, 60, 80 or 100%. The more the percentage is, the less strict it is in achieving the movement/range of movement during the game. Press 'Done' when finished.

7. Click on Easy, Medium or Hard according to the level you think your participant can perform (Start with Easy if it is the first time and you can change it to medium or hard if needed the next time).

8. Hold the sensors in your palm and move it in a figure of eight pattern for 10-15 sec to calibrate the sensors. Attach the sensor on the body part of the participant using straps so that the movement is created on the screen. The sensors are usually strapped on the body parts on either of side of the joint that is producing the movement for knee and ankle. For hip abduction, one sensor can be placed on the ankle and other on the floor towards which abduction movement is done. For hip flexion and extension, one sensor is strapped to the trunk and the other on the thigh.

9. This will take you to 'Position Set up' screen to set up the start and end position of the range of movement that you want to train.

10. Click on Automatic or Manual mode.

11. If you click on Automatic mode, the iPad will automatically pick up the joint range of the movement that you want to train when you ask the participant to do that movement. Ask the participant to do the movement many times until a circle completes on the screen, usually 5 to 8 times.

12. If you are clicking Manual, you have the independence to choose the start and end positions of the range of the movement that you want to use/train. For this, press 'Set start' and ask the participant to move through the range you want and then press 'Set End' when you want to stop or when the patient stops. This will set the start and end range of the movement that you are intending to train.

13. After the circle forms, you need to wait for the red button on the horizontal white line to move freely as the participant moves through the range. Until then keep doing the movement a few more times. Sometimes there can be a small delay.

14. After the red dot starts moving, press Play.

15. This will start the game and when the participant moves through the range that you have set, they play the game that you have chosen. Most games last a minute and counts down from 60 sec. The countdown time can change according to the game. The

repetitions come on the screen**. Please record the repetition and time played in the technology recording sheet and keep it safe in the AMOUNT2 folder under each subject ID.** There is a pause button and play button if you need to pause and then play. The game can be exited using the exit button on the top right-hand corner of the screen. You can also press 'Back' and go back to recalibrate the sensors. Sometimes, if the program hangs, you might have to trouble shoot by exiting the game and starting over again.

16. The game can be continued unlimited times by either waiting for the game to re-start on its own after certain seconds depending on the game or by pressing the restart button.

17. You can progress by choosing medium or hard in each game if you think your participant requires more challenging games. This can be done by going 'Back' to the 'Prescription' window and choosing 'Medium' or 'Hard' levels.

18. You can 'Create' as many games as you want for a participant, and it gets saved under their ID when you open each game.

## Playing a game subsequently:

Once the exercises are created and saved for a participant either 'By Workout or 'By Joint', it is easy to come back and choose an exercise/movement and play a game by using the following instructions:

1. Choose a game app that you want your participant to play like Jumpy Rabbit, Basketball, Steelman, Endless racing, Racoon rescue, Flying rocket, Sliding Santa, etc. Please refer to the table at the end of this section for the details on each game.

2. Log in into the game by using the Username: ________________and Password: _______________

3. Enter the User name: _______________ and Password: __________________

4. Click on the Participant ID of the participant and then click Play.

5. Choose and click on one of the exercises that you have already prescribed.

6. Click on Easy, Medium or Hard according to the level you think your participant can perform (Start with Easy if it is the first time and you can change it to medium or hard if needed).

7. Hold the sensors in your palm and move it in a figure of eight pattern for 10-15 sec to calibrate the sensors. Attach the sensor on the body part of the participant using straps according to the movement you are training as described previously.

8. ‘Position Set up’ screen is used to set up the start and end position of the range of movement that you want to train.
9. Click on Automatic or Manual mode.
10. If you click on Automatic mode, the iPad will automatically pick up the joint range of the movement that you want to train when you ask the participant to do that movement. Ask the participant to do the movement many times until a circle completes on the screen, usually 5 to 8 times.
11. If you are clicking Manual, you have the independence to choose the start and end positions of the range of the movement that you want to use/train. For this, press ‘Set start’ and ask the participant to move through the range you want and then press ‘Set End’ when you want to stop or when the patient stops. This will set the start and end range of the movement that you are intending to train.
12. After the circle forms, you need to wait for the red button on the horizontal white line to move freely as the participant moves through the range. Until then keep doing the movement a few more times. Sometimes there can be a small delay.
13. After the red dot starts moving, press Play.
14. This will start the game and when the participant moves through the range that you have set, they play the game that you have chosen. Most games last a minute and counts down from 60 sec. The countdown time can change according to the game. The repetitions come on the screen**. Please record the repetition and time played in the technology recording sheet and keep it safe in the AMOUNT2 folder under each subject ID.** There is a pause button and play button if you need to pause and then play. The game can be exited using the exit button on the top right-hand corner of the screen. You can also press ‘Back’ and go back to recalibrate the sensors. Sometimes, if the program hangs, you might have to trouble shoot by exiting the game and starting over again.
15. The game can be continued unlimited times by either waiting for the game to re-start on its own after certain seconds depending on the game or by pressing the restart button.
16. You can progress by choosing medium or hard in each game if you think your participant requires more challenging games. This can be done by going ‘Back’ to the ‘Prescription’ window and choosing ‘Medium’ or ‘Hard’ levels.

## Choosing a game to play to train the movement:

| Game | Mobility activity | Game length | Description | Recording | Progress |
|---|---|---|---|---|---|
| Jumpy Rabbit | **-Sitting balance**<br>**-Sit-to-stand**<br>**-Static standing balance**<br>**-Toe taps**<br>**-Step ups**<br>**-AROM**<br>**-Strength training**<br>**-Walking** | Game stops when the fox eats the rabbit | The aim of the player is to make the rabbit jump as fast as one can to escape the chasing fox. Each assigned movement make the rabbit jump and each jump is recorded as a repetition. If there are four sensors available, it can be used as a two-player game and can be more fun | Record the number of repetitions that come up on the screen | Medium and Hard levels -The speed at which the fox chases the rabbit increases |
| Basketball | **-Sitting balance**<br>**-Sit-to-stand**<br>**-Static standing balance**<br>**-Toe taps**<br>**-Step ups**<br>**-AROM**<br>**-Strength training**<br>**-Walking** | Game stops after 60 sec countdown | The aim of the player is to bounce the ball into basket and score a shot with each assigned movement. Each shot is a repetition. | Record the number of repetitions that come up on the screen | Medium level - the ball has to go past a moving hand that tries to block the ball.<br>Hard level - the hand tries to block the ball as well as the basketball loop moves from one side to the other |
| Sliding Santa | **-Sitting balance**<br>**-Sit-to-stand**<br>**-Static standing balance**<br>**-Toe taps**<br>**-Step ups**<br>**-AROM**<br>**-Strength training** | Game stops when Santa crashes into barriers | The aim of the player is to slide the Santa and collect presents on the way with each assigned movement. Each movement slides the Santa left or right. The score represents the number of presents collected | Record the active time in minutes played or the physiotherapist record the number of repetitions | Medium and Hard levels- The speed at which Santa slides increases with harder levels |

| | | | | | |
|---|---|---|---|---|---|
| Racoon Rescue | **-Sitting balance**<br>**-Sit-to-stand**<br>**-Static standing balance**<br>**-Toe taps**<br>**-Step ups**<br>**-AROM**<br>**-Strength training** | Games stops when all raccoon babies are saved | The player is required to save all raccoon babies by throwing/shooting the bubble that raccoon is holding. By doing each assigned movement, the shooting target is moved from one end to the other and when the colour of the balloon that the raccoon holds matches the colour of the target, the physiotherapist presses the shoot button.The bubbles burst and the baby is saved. Depending on how many bubbles burst, the participant scores a point. | Record the number of minutes the game was actively played and the score | With each win, you are taken to a higher level. With each higher level there are more bubbles to pop and lesser bubbles to throw and therefore lesser turns. |
| Rolling Ball | **-Sitting balance**<br>**-Sit-to-stand**<br>**-Static standing balance**<br>**-Toe taps**<br>**-Step ups**<br>**-AROM**<br>**-Strength training** | Games stops when the ball rolls off the platform or hit a red block in the way. | The aim of the player is to keep the ball rolling on the platform and collect diamonds as it rolls. | Record the number of minutes the game was actively played and the score of diamonds collected | The game moves itself on to the next levels after every checkpoint. With each level it gets harder with ball rolling faster through more slanted slopes |
| Endless Racing | **-Sitting balance**<br>**-Sit-to-stand**<br>**-Static standing balance**<br>**-Toe taps**<br>**-Step ups**<br>**-Walking**<br>**-Strength training** | Game stops when the fuel runs out | The aim of the player is to collect coins on the way by moving the car left and right with each assigned movement. The player can collect coins, petrol, coin magnet, boost. | Record the number of minutes the game was actively played, and the number of coins collected | There is only one level |

| | | | | | |
|---|---|---|---|---|---|
| | | | | | |
| **Steel man** | **-Sitting balance**<br>**-Sit-to-stand**<br>**-Static standing balance**<br>**-Toe taps**<br>**-Step ups**<br>**-AROM**<br>**-Strength training** | Game stops when the time runs out. Easy level is 60 sec, medium is 120 sec and Hard is 180 sec | The aim of the player is to take the steelman between the obstacles by performing the assigned movement and collect diamonds on the way. The number of diamonds collected get shown. Record the number of minutes the game was actively played, and the number of diamonds collected. | Record the number of minutes the game was actively played, and the number of diamonds collected | Medium and Hard levels- The speed at which the steelman flies increases with harder levels |
| **Flying Rocket** | **-Sitting balance**<br>**-Sit-to-stand**<br>**-Static standing balance**<br>**-Toe taps**<br>**-Step ups**<br>**-AROM**<br>**-Strength training** | Game stops when the rocket fall | The aim of the player is to keep the rocket flying. The assigned movement moves the rocket up and down and the player collects stars, astronauts, aliens and scores a point each time. | Record the number of minutes the game was actively played and the score | Medium level: the speed increases and there are large asteroids to collect. Hard level: The rocket changes to a spaceship and is very fast and the player has to work very hard to keep the spaceship from falling |
| **Hockey** | **-Sitting balance**<br>**-Sit-to-stand**<br>**-Static standing balance**<br>**-Toe taps**<br>**-Step ups**<br>**-AROM** | Game stops when the computer loses the game | The aim of the player is to bounce the ball back to the computer using a space bar. The assigned movement moves the space bar up and down on which the ball bounces. | Record the number of minutes the game was actively played and | Medium and Hard levels- The speed at which the ball bounces increases with increase in levels |

| | | | | | |
|---|---|---|---|---|---|
| | **-Strength training** | | | the score | |
| **Tetromino** | **-Sitting balance**<br>**-Sit-to-stand**<br>**-Static standing balance**<br>**-Toe taps**<br>**-Step ups**<br>**-AROM**<br>**-Strength training** | Game stops when the ball drops off the platform that it is balancing on or when the 120 sec time finishes | The aim of the player is to maintain the ball on the platform as well as not get hit by any obstacles. Each movement assigned is counted as a rep. The player can collect diamonds which adds up as separate points. | Record the number of repetitions and number of minutes actively played | Medium and Hard levels- the speed at which the obstacles and diamond flies in increases with harder levels. |
| **Music Smash** | **-Sitting balance**<br>**-Sit-to-stand**<br>**-Static standing balance**<br>**-Toe taps**<br>**-Step ups**<br>**-AROM**<br>**-Strength training** | Game stops if 3 min timer finishes or if the player does not smash the objects with the bat for more than 3 times | The aim of the player is to smash the objects with a baseball bat as it comes flying past it. The player should also avoid hitting detonators. | | Medium and Hard levels- the speed at which the objects come flying past increases with harder levels |
| **Lusio Tetrix** | **-Sitting balance**<br>**-Sit-to-stand**<br>**-Static standing balance**<br>**-Toe taps**<br>**-Step ups**<br>**-AROM**<br>**-Strength training** | Game stops when the blocks fill up the area | The aim of the player is to stack the blocks to fill up the space. With each assigned movement, the blocks move from left to right. When each block touches the base, the player earns 10 points. | Record the number of repetitions and the minutes actively played | Medium and Hard levels- the speed at which the blocks come down increases with harder levels |

| | | | | | |
|---|---|---|---|---|---|
| **Shape Change** | **-Sitting balance**<br>**-Static standing balance**<br>**-Toe taps**<br>**-Step ups**<br>**-AROM**<br>**-Strength training** | Game ends when you lose all 3 lives | The aim of the player is to have different shapes pass through its shaped hole. Each time it passes the right hole, the player scores a point. Each time it goes through the wrong hole, player lose a life. Lives can be regained by collecting diamonds | Record the number of repetitions and the minutes actively played | There is only one level |
| **Poppop** | **-Sitting balance**<br>**-Sit-to-stand**<br>**-Static standing balance**<br>**-Toe taps**<br>**-Step ups**<br>**-AROM**<br>**-Strength training** | Game ends when the ball falls off the ledge | The aim of the player is to maintain the ball on the platform as well as to let it move into new platforms and targets with each movement. | The repetitions are counted by the game and shown as total reps after the game finishes | There are more than 4 levels of game with difficulty increasing with each level. |
| **Red Runner** | **-Toe taps**<br>**-Step ups**<br>**-Walking** | Game finishes after 10 min countdown or if the red runner hits an obstacle | The aim of the player is to walk the red runner with each movement assigned. If you press the narrow arrow, red runner jumps high and if you press the wider arrow, it jumps wider. | The distance walked is recorded | Medium and hard levels have more harder obstacles |
| **Mountain Bike** | **-Sit-to-stand**<br>**-Toe taps**<br>**-Step ups**<br>**-Walking**<br>**-AROM** | Game finishes when the bike crosses the finish line | The aim of the player is to cycle the bike with each assigned movement through the obstacles. The bike jumps if the jump button is pressed. Completion of each level unlocks the next level. | Record the number of minutes actively played | There are 10 levels of game and the obstacle course become harder with each high level |

| | | | | | |
|---|---|---|---|---|---|
| | **-Strength training** | | | | |
| **Road Bomber** | **-Sitting balance**<br>**-Sit-to-stand**<br>**-Static standing balance**<br>**-Toe taps**<br>**-Step ups**<br>**-AROM**<br>**-Strength training** | Game finishes after 60 sec count down | The aim of the player is to bomb the tankers on the road which gives points according to the number of bombs. The assigned movement make bomber to go left and right to target the tankers. | Record the number of minutes actively played and the repetitions of movement. | Medium and Hard level- Bomber flies past faster and there are more tankers to bomb. |
| **Kango** | **-Sitting balance**<br>**-Sit-to-stand**<br>**-Static standing balance**<br>**-Toe taps** | Game finishes when 2 minute countdown finishes | The aim of the player is to follow the Kangaroo and collect rain drops in a container. The assigned movement makes the container move left and right | Record the number of minutes actively played and the repetitions of movement. | Medium and Hard- the speed at which the rain drops gets faster. |

## Placement of sensors:

The sensor placement depends on the movement that you want to train. Usually, the sensors are placed on the body parts on either side of the joint that is producing the movement, for e.g. in order to train ankle dorsiflexion, you can have one sensor on the dorsum of the foot and the other on the anterior aspect of the lower leg. Sometimes, you will have to keep one sensor on the ground or on an external area for the sensors to pick up the movement that you want to train, for e.g. If you want to train weight shift in standing, you can do that by having one sensor on the lateral aspect of ankle or thigh and the other on the floor beside the ankle, whereby the sensors pick up the

movement during the weight shift by the distance the sensors move from each other. So as long as the sensors pick up the movement, the placement of sensors is upon your discretion. Please see the table below for some common sensor placements:

| Movement | Placement of sensors | Movement | Placement of sensors |
|---|---|---|---|
| 1. Sitting balance Training | Sensor 1: Strap around the wrist<br>Sensor 2: Place near the target | 5. Toe taps | Sensor 1: Strap around the thigh<br>Sensor 2: Strap around the lower leg |
| 2. Sit-to-Stand Training | Sensor 1: Strap around the thigh<br>Sensor 2: Strap around the lower leg | 6. Step ups | Sensor 1: Strap around the thigh<br>Sensor 2: Strap around the lower leg |
| 3. Weight shift in standing | Sensor 1: Strap around the lateral aspect of ankle<br>Sensor 2: Place it on the floor in line with the sensor 1 about 10 cm away | 7. Walking | Sensor 1: Strap around the thigh<br>Sensor 2: Strap around the lower leg |
| 4. Reaching in standing | Sensor 1: Strap around the wrist<br>Sensor 2: Place near the target | 8. Strength training | Sensor 1: Strap on the limb proximal to the joint<br>Sensor 2: Strap on the limb distal to the joint |

## *PTX – PhysioTherapyeXercises*

PTX or physiotherapyexercises.com is a free website where you can create exercise programs for your participants. There are 11 languages to choose from. These exercises can be sent to the participant's mobile phone number or to their email address. When the participant clicks the link, it saves and opens as a 'PTX' icon on the phone with the exercises that you have prescribed. You can also provide a printed copy of the exercises to the participant if needed. An exercise schedule for the week can be attached to the exercises. The website provides the compliance data of the participants to the prescribing physiotherapist.

### Getting started

- Use the web address https://www.physiotherapyexercises.com in the Internet explorer or Google Chrome search engines to reach the home page of the website. A prompt comes up on the site asking if you would like to install it as an app on your desktop. You can either choose to install the app or work directly from the site. On the Home page, on the left-hand top corner choose the language as needed. Default language is English, there are 11 other languages to choose from.

- Create a new account or log in to your account if you already have an account. By creating an account, you can save all the exercises in your account, review and change them as needed. You need a login name, a password and an email address to create an account. You will receive an email to verify your email account. Please check your 'junk mail' if you do not see it in your inbox. Once the email is verified, the account is established.

- You can also choose to not log in, search for exercises, select and make a booklet and send it to the participant. But you can't print the exercises unless you log in. it is free to make an account.

### Selecting and creating an exercise booklet:

- Search for the exercises by clicking the search button on the left lower corner of the home screen. You can narrow the search for a particular exercise from more than 1000 exercises from the website using the 8 categories provided. Categories include headings like 'Condition', 'Exercise difficulty', 'Equipment available', etc. If you click on these headings, they open up various options to select from, for exam, If you click 'Conditions', you can choose the condition that your participant has like stroke, multiple sclerosis, spinal cord injury etc

- Under the heading 'Text to display with exercise images', you have 7 different options of text like 'Therapist's aim', 'client's' aim, etc. to choose from and display along with the prescribed exercises.
- Once you select the options that you would like to add, click on 'Exercises' on left lower corner of the screen to display images of all the exercises related to the options that you have chosen.
- Hover the mouse on the exercise that you would like to select, and it will give you details of the exercises. Click on the image if you would like to select any particular exercise and click again to deselect if you do not want to. You have also option on the right top corner to 'select all' or 'deselect all'.
- Once you have selected the exercises, click 'Wizard' on lower left corner and follow the prompts to send the exercise as a link to mobile/email, or as a word or pdf document to email, which can be printed.
- If you had chosen to send a link to client's mobile, you have to provide the country code and the mobile number or you can send the link to the client's email.
- You also have various choices when you follow the wizard like 'duration'- how many weeks program you are prescribing, 'display image/text', 'add your contact details', recording allowed or not to show if the client has been compliant with the exercises which the physiotherapist can monitor if the client gives consent to it.
- At any point you are unsure of what to do, you can click on the tutorial, and it will guide you.
- If you log in, the exercises that you create get saved.

| PTX APP | | |
|---|---|---|
| **Program** | **Exercises** | **Modifications/ feedback** |
| **Preparation for standing up** | 1. Flexing the hips in sitting and sliding arms forward on a table to a target<br>2. Bending the knee in sitting<br>3. Pushing down through the leg in sitting<br>4. Sitting and reaching quickly<br>5. Sitting and reaching to the affected side<br>6. Standing up and sitting down from a high surface | Edit or replace exercises, take video of participant doing exercise/ exercise completion sent weekly to therapist, fb by therapist over phone, graphs. |
| **Standing up** | 1. Sitting forward<br>2. Moving the shoulders forwards in sitting to a target<br>3. Reaching to the side when standing up<br>4. Standing up and sitting down to a knee cue<br>5. Standing up and sitting down with hand support nearby<br>6. Standing up and sitting down from a dining table<br>7. Standing up and sitting down when holding an object<br>8. Standing up with one leg on a block<br>9. Standing up and walking | Edit or replace exercises, take video of participant doing exercise/ exercise completion sent weekly to therapist, fb by therapist over phone, graphs. |
| **Maintaining a standing position** | 1. Hip extension in standing against a wall<br>2. Bilateral squat to a target in standing<br>3. Standing and leaning forwards and backwards<br>4. Transferring weight laterally in standing against two walls<br>5. Standing and turning with feet close together<br>6. Standing in tandem stance<br>7. Standing on one leg with support | Edit or replace exercises, take video of participant doing exercise/ exercise completion sent weekly to therapist, fb by therapist over phone, graphs. |

| | | |
|---|---|---|
| **Reaching in standing** | 1. Reaching from side to side in standing<br>2. Standing and reaching<br>3. Standing and reaching to the side<br>4. Standing and reaching from the floor to above the head<br>5. Stepping forward to reach for an object | Edit or replace exercises, take video of participant doing exercise/ exercise completion sent weekly to therapist, fb by therapist over phone, graphs. |
| **Stepping in standing** | 1. Marching on the spot<br>2. Maintaining single leg stance while touching the other foot on a block<br>3. Stepping<br>4. Stepping forwards over an obstacle with hand support nearby<br>5. Stepping forward to reach for an object<br>6. Stepping sideways against a wall<br>7. Stepping to targets with hand support nearby<br>8. Maintaining single leg stance while stepping backwards with the other leg | Edit or replace exercises, take video of participant doing exercise/ exercise completion sent weekly to therapist, fb by therapist over phone, graphs. |
| **Changing directions while walking** | 1. Stepping sideways against a wall<br>2. Stepping to targets with hand support nearby<br>3. Swivelling on the balls of the feet<br>4. Turning around on the spot<br>5. Walking in a figure-of-eight<br>6. Standing up and walking<br>7. Rolling the foot on a ball while standing<br>8. Walking and dribbling a ball around objects | Edit or replace exercises, take video of participant doing exercise/ exercise completion sent weekly to therapist, fb by therapist over phone, graphs. |
| **Climbing stairs** | 1. Raising the leg onto a block<br>2. Stepping up onto a block<br>3. Maintaining single-leg-support while stepping up and down on a stairs<br>4. Stepping down from a block to a knee cue<br>5. Moving the leg back and forth while stepping | Edit or replace exercises, take video of participant doing exercise/ exercise completion sent weekly to therapist, fb by therapist over phone, graphs. |

| | | |
|---|---|---|
| | 6. Walking up stairs<br>7. Walking down stairs | |
| **Physical activity throughout the day** | 1. Walking a set distance within a set time<br>2. Marching on the spot<br>3. Walking on slopes<br>4. Walking on uneven ground<br>5. Standing up and walking | Edit or replace exercises, take video of participant doing exercise/ exercise completion sent weekly to therapist, fb by therapist over phone, graphs. |
| **Running, hopping and jumping** | 1. Jumping forwards over lines<br>2. Jumping off a low object<br>3. Leaping from a standing start and landing on one leg | Edit or replace exercises, take video of participant doing exercise/ exercise completion sent weekly to therapist, fb by therapist over phone, graphs. |

| | | |
|---|---|---|
| | 4. Leaping sideways<br>5. Hopping on the spot<br>6. Stepping onto a stool off tip toes<br>7. Swinging the foot quickly up to a target<br>8. Running between lines | |
| **Low difficulty mobility** | 1. Ankle dorsiflexor strengthening in sitting without weights<br>2. Ankle plantarflexor strengthening in sitting without weights<br>3. Bending the knee in sitting<br>4. Hip flexor strengthening in sitting<br>5. Single leg hip extensor strengthening in supine<br>6. Hip abductor strengthening in sidelying without weights<br>7. Bilateral squat in standing<br>8. Standing with the feet together<br>9. Standing in semi-tandem stance<br>10. Reaching while seated | Edit or replace exercises, take video of participant doing exercise/ exercise completion sent weekly to therapist, fb by therapist over phone, graphs. |

| | | |
|---|---|---|
| | 11. Standing up and sitting down from a high surface | |
| **Medium difficulty mobility** | 1. Knee control in standing<br>2. Lifting an object from the floor to the table<br>3. Standing up and sitting down with hand support nearby<br>4. Hip abductor strengthening in standing against a wall<br>5. Maintaining single-leg stance while stepping backwards with the other leg<br>6. Maintaining single-leg stance while touching the other foot on a block<br>7. Moving from the heels to the toes in standing<br>8. Standing and picking up an object off the floor<br>9. Standing and turning with one leg forward<br>10. Stepping forwards to reach for an object<br>11. Stepping to targets with hand support nearby | Edit or replace exercises, take video of participant doing exercise/ exercise completion sent weekly to therapist, fb by therapist over phone, graphs. |
| **High difficulty mobility** | 1. Getting up from the floor<br>2. Standing and picking up an object off the floor with both hands<br>3. Standing up and sitting down from a low stool<br>4. Lowering and raising from a block<br>5. Raising and lowering from a block | Edit or replace exercises, take video of participant doing exercise/ exercise completion sent weekly to therapist, fb by therapist over phone, graphs. |

| | |
|---|---|
| | 6. Single leg heel raise<br>7. Maintaining single leg stance while rolling a ball around an obstacle<br>8. Braiding<br>9. Walking a set distance within a set time<br>10. Walking backwards<br>11. Walking on slopes<br>12. Walking up a kerb<br>13. Walking while performing multiple tasks |
| **Skill** | 172 exercises to choose from |
| **Strength** | 69 exercises to choose from |
| **Stretch** | 11 exercises to choose from |

# *CLOCK YOURSELF*

Clock yourself is a reaction time training app that may help to improve balance and cognition. Its aim is to simultaneously train your body and brain. The exercises are designed around an imaginary clockface that the participant has to visualise on the floor beneath their feet when they are standing. The
instructions for the exercises are provided by the 'Clock yourself' app through audio from the app, which the participant has to follow.

## Getting started

- Download and install the 'Clock yourself' app on a tablet or the participant's phone from the Apple store for iPhone apps or Google play for Android apps.

## Selecting the levels

There are 6 levels of progression to choose from when you open the 'Clock yourself' app. Choose the level that would suit the participant according to the assessment. The levels are as listed below and it goes from simpler to more harder levels from top to bottom.

| | |
|---|---|
| **1. Simple Colours:** This is the first level and simplest one. The participant stands in a space with no obstacles around. Activity is to imagine four colours (yellow, green, red, and blue) under you and take steps to one of four colours as prompted by the app when you press start. In the options you can choose to change speed and duration. | **2. Simple Clock:** The participant stands in a space with no obstacles around. Activity is to imagine clockface under you and take steps in 12 directions of time as prompted by the app when you press start. In the options you can choose to change the clock shape, language, speed and duration. |
| **3.Brain Games:** The participant stands in a space with no obstacles around. Activity is to imagine a calendar on a clockface under you and take steps in 12 directions of months as prompted by the app when you press start. In the options you can choose to change the clock face to minutes, doubled numbers, star signs, animals, periodic table, and symbols. You can also change, language, speed and duration. | **4.Coordination:** The participant stands in a space with no obstacles around. Activity is to imagine a clockface under you and take steps as well as do extra activities. The extra activities include centre touchdown, bicep curls, chest press. In the options you can also choose to change the clock face, language, speed and duration. |

| | |
|---|---|
| **5.Complex Combination:** The participant stands in a space with no obstacles around. Activity is to imagine a clockface under you and take steps as prompted by the app to a clock hour, month, number, or minutes including the choice of extra activities with it. In the options you can. choose to change the clock face to multiple options of minutes, doubled numbers, star signs, animals, periodic table, and symbols. You can also choose to add extra activities of centre touchdown, bicep curls, chest press and also change, language, speed and duration. | **6.Athletic Agility:** The participant stands in a space with no obstacles around. Activity is to imagine a clockface under you and take steps with both feet or as prompted by the app to a clock hour along with extra activity of touch down. You can also change, language, speed and duration. |

## Using the App:

- Have an open space for the participant to stand and perform stepping where they can imagine a clockface under their feet. Open the app and choose the level that you want to use. This opens a clockface with two feet in the centre. In the bottom end of the screen in the middle is a green start button. Increase the volume of the device and press the start button. It will call out the number that the participant has to step on the clockface. Press the pause button to stop if needed. The 'Duration' on the bottom right will start counting down from 5 minutes when you start the program. You can choose to increase to a maximum of 10 min or decrease to a minimum of 30 seconds by clicking 'Duration' button on the bottom right. You can decrease or increase the speed of the exercises by clicking on 'Speed' button on the bottom left.

- Detailed instructions on how to use the app are also provided under 'Instructions' on the left bottom part of the screen.

- 'Options' on the right bottom provides you with options of choosing the clock shape, language, duration, and speed. Under clock shape, you can choose the full clockface, left half, right half, bottom half etc. Under 'Language', you can choose from 7 languages.

## Exercising using the app:

- To start exercising, the participant stands in an open space where large steps can be taken in all directions. Visualise a large clockface under the floor beneath and stand in the middle of the clock. The 12 imaginary numbers are now the coordinates and the participant should not have to look at the screen to know where they are. When the number is heard, the participant has to step on it and transfer weight to that foot and step back to the centre after each step. As the reaction gets faster, the speed and difficulty are increased.

- If the participant needs to hold on in order to participate, then a half face or a bottom face clockface can be opted under the 'Options'.

## *STEPWATCH*

It is a rehabilitation device/monitoring system which involves a 3-inch sensor that can be easily worn around the ankle using a Velcro band. It measures the number of steps, cadence and active time in minutes.

### Getting started with StepWatch:

The therapist will need a fully charged StepWatch and an AMOUNT 2 study iPad to use it on a participant.
The participant will need their iPhone or an iPad if available in order to download an App to track their own progress.

### Open the following app on the AMOUNT 2 iPad:

**Modus Health CC Clinic for staff:** This App downloads the number of steps the participant takes, helps the therapist to set up a goal of number of steps per day for the participant to work towards according to the participant's capability, and helps send messages to motivate the participant to achieve their goal.

### Download and open the following app on participant phone or iPad:

**Modus Health CC Companion for participants:** This App is connected to the StepWatch and downloads the number of steps the participant takes onto the participant's iPad or iPhone and therefore helps the participant to keep track of his/her progress and attain their goals set by the therapist.

### Using Modus Health CC Clinic for staff:

- Select the team
- Select a team member: choose your name or colleague's name from the list and type in the corresponding pin that is provided to the team
- This will take you to the mobility triage report. Note this shows you the list of active participants and it shows you the data from the day before so if the participant is new, there will not be any data for that participant here.
- Menu- on the bottom of this page there are 3 main icons:
    - Home: this is the page you currently are on. You can connect StepWatches, start a PT session, checkout participant, and send messages to the participant.

- Participants: This is where all the participants are listed with their data (old and current). You can press filters to filter participants for a particular time period, or according to goals met (yes/no), and enrolled (current participants). If you click the participant name, it opens their stored information.
- Messages: Here you can send messages to participant's mobile or ipad app.

- **Enrolling a new participant:**
  - Go to home page, select Connect StepWatch
  - StepWatches that are within the Bluetooth connection range will show up on the iPad screen. Select the StepWatch that you want to connect to from the list. If the StepWatch do not come up, then place the StepWatch facing down on the lined space shown on the iPad. If the StepWatch still does not connect, it is probably because it doesn't have enough battery left, so please place it on the charger to get it charged before using it.
  - To set up a new participant select "New participant".
  - Fill in the participant's name (First name & initial of surname or subject number assigned). Then select height, select gait according to the level of impairment (no, slightly, severely). Set goals: good to start low e.g., 500 or 1000 steps per day, minutes active-30 mins, peak steps/min-40-60. You can modify the goal any time after you verify the participant's step count by doing a step count test. This is done by asking the participant to walk 10 steps while you press the start button and stop the button when they are done. This will calculate number of steps and peak steps per minute. You can repeat the step count again if you think it was not done well previously.
  - A QR code will come up on the iPAD. This is when you set up the participant companion app either on their iPhone or iPad. Open the participant companion app and scan the QR code. If the participant is not ready to set up this app, you can take a photo of the QR code to use it later and then press "skip".
  - Once this is done, the participant is linked to the app and is set up. The step watch is now attached to the ankle using a Velcro strap and it will start collecting the step count when the participant starts to walk.
  - If you are assigning the StepWatch to a New Participant and if it was already assigned to another participant, make you select "Checkout" first. When you do this the previous patient is checked out and the StepWatch goes into a storage mode to save battery. You need to wake up the StepWatch to proceed. To do this, you place the StepWatch on the charger for ≥ 10secs. After which you go back to the iPad and select a new participant to assign the StepWatch to the participant.
  - If you are setting up a "checked out" returning participant, then select "returning participant".
- **Updating participant data onto the iPad:**
  - Open the Modus clinic CC app for staff. Select "Connect StepWatch". The active StepWatches will appear, select the one you want to update from the list. Select "Update Step Data". A green message will appear below letting you know that the data has been uploaded successfully. You can then go to the participant menu, select the participant from the list and see the updated data. Note:

If your participant has their iPad with them and the StepWatch is connected to that, then your iPAD won't update the data at that time.

- **Recording activity in a Physiotherapy session:**
    - This can only be done from Modus clinic cc app for staff. On the home page, select "Start PT session". It takes you to a Physical Therapy screen and you can select "Start", "Pause" and "Stop". It will then record steps and active time and peak steps/min during the therapy session (and it will be added to the total values). Note: Using therapy mode will use more battery power (the battery % is shown on this screen). You will need to charge it more regularly e.g., every 5 days if using this mode each day.
- **Sending a message to the participant:**
    - This can be done from the home page. Select "Compose". It will ask you which participant you wish to send messages to from dropdown list. Select the participant and write the message and send. If a participant has sent you a message the message icon at the bottom of the screen will be blue colour. You can select and see what messages you have received.
- **Updating participant goals:**
    - Select participant name from participants menu. You can only update goals for active participants. You will see it written in blue at the top "Update Goals". The 3 goal options come up and you can modify. The participant will then be notified on their app that their goals have been updated. You might want to send them a message before updating e.g. great work, you have achieved all your goals so I am going to make them more challenging.
- **Recording participant's Step data:** Please enter the step counts for each day in the Digital Devices practice sheet provided.

# INPATIENT PRACTICE SHEET

<table>
<tr><td>INPATIENT PRACTICE SHEET</td><td></td><td></td><td></td><td></td><td></td><td></td></tr>
<tr><td>Session duration</td><td>45 min</td><td>55 min</td><td>45 min</td><td>63 min</td><td>65 min</td><td></td></tr>
<tr><td>MOBILITY LIMITATION</td><td>2</td><td>2</td><td>1</td><td>1</td><td>1</td><td></td></tr>
<tr><td>TECHNOLOGY</td><td>3</td><td>3</td><td>4</td><td>4</td><td>4</td><td></td></tr>
<tr><td>GAME</td><td>Weight shift</td><td>Weight shift</td><td>Sit to stand</td><td>Sit to stand</td><td>Sit to stand</td><td></td></tr>
<tr><td>EXERCISE LEVEL</td><td>L2; Boundary</td><td>L2; Boundary</td><td>bed ht:52cm; L1</td><td>bed ht:50cm; L3</td><td>Bedht:50cm;L10</td><td></td></tr>
<tr><td>SETS / REPS / DURATION (min)</td><td>2 | 10 | 5</td><td>2 | 10 | 5</td><td>2 | 25 | 6</td><td>2 | 24 | 6</td><td>2 | 65 | 14</td><td></td></tr>
<tr><td>SCORE / CUES</td><td></td><td></td><td>450</td><td>480</td><td>1720</td><td></td></tr>
<tr><td>SET UP | USE | RECORD = I/P/A</td><td>A | P | A</td><td>A | P | A</td><td>A | A | A</td><td>A | P | A</td><td>A | A | A</td><td></td></tr>
<tr><td>MOBILITY LIMITATION</td><td>2</td><td>2</td><td>4</td><td>4</td><td>4</td><td></td></tr>
<tr><td>TECHNOLOGY</td><td>2</td><td>3</td><td>4</td><td>4</td><td>4</td><td></td></tr>
<tr><td>GAME</td><td>Penguin slide</td><td>Instability</td><td>Side walking</td><td>Side walking</td><td>Lunges</td><td></td></tr>
<tr><td>EXERCISE LEVEL</td><td>Beginner</td><td>Level 2; hold 5s</td><td>L1</td><td>L2</td><td>Level 20</td><td></td></tr>
<tr><td>SETS / REPS/ DURATION (min)</td><td>3 | | 10</td><td>10 | | 12</td><td>2 | 20 | 6</td><td>2 | 25 | 8</td><td>4 | 25 | 10</td><td></td></tr>
<tr><td>SCORE / CUES</td><td>35,30,40</td><td></td><td>400</td><td>500</td><td>2860</td><td></td></tr>
<tr><td>SET UP | USE | RECORD = I/P/A</td><td>A | A | A</td><td>A | P | A</td><td>A | A | A</td><td>A | A | A</td><td>A | A | A</td><td></td></tr>
<tr><td>MOBILITY LIMITATION</td><td></td><td>2</td><td>4</td><td>4</td><td>4</td><td></td></tr>
<tr><td>TECHNOLOGY</td><td></td><td>2</td><td>4</td><td>4</td><td>4</td><td></td></tr>
<tr><td>GAME</td><td></td><td>Penguin slide</td><td>Lunges</td><td>Lunges</td><td>Dynamic balance</td><td></td></tr>
<tr><td>EXERCISE LEVEL</td><td></td><td>Beginner</td><td>L20</td><td>L20</td><td>L6 random</td><td></td></tr>
<tr><td>SETS / REPS / DURATION (min)</td><td></td><td>3 | | 10</td><td>6 | 15 | 8</td><td>4 | 15 | 8</td><td>2 | | 8</td><td></td></tr>
<tr><td>SCORE / CUES</td><td></td><td>35,30,40</td><td>2340</td><td>1890</td><td>840</td><td></td></tr>
<tr><td>SET UP | USE |RECORD = I/P/A</td><td></td><td>A | A | A</td><td>A | P | A</td><td>P | P | A</td><td>A | A | A</td><td></td></tr>
<tr><td>COMMENTS (eg. date start teaching; date indep achieved)<br>Step count:</td><td>1st session:<br>Intervention planning sheet; demonstration</td><td>Set up fitbit + fitbit App on iPAD</td><td>Review steps on iPAD<br>840</td><td>Review steps on iPAD<br>658</td><td>Review steps on iPAD<br>1620</td><td></td></tr>
<tr><td></td><td></td><td></td><td></td><td></td><td></td><td></td></tr>
</table>

| INPATIENT PRACTICE SHEET | Monday 27/4 | Tuesday 28/4 | Wednesday | Thursday | Friday | Weekend |
|---|---|---|---|---|---|---|
| **Session Duration** | | cont. | cont. | cont. | cont. | |
| **MOBILITY LIMITATION** | | 2 | 3 | 3 | 4 | |
| **TECHNOLOGY** | | 2 | 1 | 1 | 5 | |
| GAME | | Table tilt | Wall breaker | Wall breaker | Stepping grid | |
| EXERCISE LEVEL | | Beginner | easy | easy | 4 steps | |
| SETS / REPS / DURATION (min) | | \| 5 \| 11 | \| 5 \| 10 | \| 6 \| 12 | 20L \| 20R \| 10 | |
| SCORE / CUES | | 40 | 543 | 620 | - | |
| SET UP \| USE \| RECORD = I/P/A | | P \| P \| A | A \| P \| A | A \| P \| A | A \| P \| A | |
| **MOBILITY LIMITATION** | | 4 | 3 | 3 | 6 | |
| **TECHNOLOGY** | | 2 | 1 | 1 | 5 | |
| GAME | | Jogging | Fruit Ninja | Super saver | Step up/ down | |
| EXERCISE LEVEL | | Beginner | | - | 10cm block | |
| SETS / REPS / DURATION (min) | | 1 \| \| 5 | 5 \| \| 10 | \| 5 \| 10 | 50L \| 50R \| 12 | |
| SCORE / CUES | | 24% | 120 points | - | - | |
| SET UP \| USE \| RECORD = I/P/A | | A \| A \| A | A \| P \| A | P \| I \| A | A \| P \| A | |
| **MOBILITY LIMITATION** | | | | 4 | 1 | |
| **TECHNOLOGY** | | | | 1 | 7 | |
| GAME | | | | 20000 Leaks | Prep for STS | |
| EXERCISE LEVEL | | | | - | 3/6 ex.'s | |
| SETS / REPS / DURATION (min) | | | | \| 4 \| 10 | \| \| | |
| SCORE / CUES | | | | 90 | Set-up & demo | |
| SET UP \| USE \| RECORD = I/P/A | | | | A \| P \| A | \| \| | |
| **COMMENTS** (eg. date start teaching; date indep achieved<br>**Step Count:** | | | | | To do Prep for STS ex over weekend In room | |

**MOBILITY LIMITATION: [1]** STANDING UP FROM CHAIR, **[2]** MAINTAINING STANDING POSITION, **[3]** REACHING WHILE STANDING, **[4]** STEPPING WHILE STANDING,
**[5]** CHANGING DIRECTIONS WHILE WALKING, **[6]** STAIR CLIMBING, **[7]** PHYSICAL ACTIVITY THROUGHOUT THE DAY
**TECHNOLOGY: [1] XBOX KINECT, [2] NINTENDO WII, [3] HUMAC, [4] FYSIOGAMING, [5] STEPPING TILES, [6] FITBIT, [7] IPAD**
