# Supplementary material for: Implementing digital devices to increase mobility training for people receiving inpatient rehabilitation: protocol for a feasibility hybrid type II randomized controlled trial
Source: Pilot Feasibility Stud. 2023 Apr 25;9:69. doi: 10.1186/s40814-023-01298-y (PMC10126551; doi:10.1186/s40814-023-01298-y)
Supplement: Supplementary file 3 — Additional file 3. Description of digital devices used in the trial for intervention participants. Name of digital devices used and their description. participants. [file 40814_2023_1298_MOESM3_ESM.pdf]

ADDITIONAL FILE 3

Description of digital devices used in this trial for intervention participants:

**Name of the digital device**	**Description**
1. Nintendo WiiFit, Nintendo, Kyoto, Japan	A commercially available device that has a balance board that provides feedback regarding the centre of pressure under the feet.
2. Xbox Kinect, Microsoft Redmond Campus, Redmond, Washington, USA	A commercially available device that uses a sensor to determine a user's movement in space.
3. Fysiogaming, Doctor Kinetic, Amsterdam, The Netherlands	A rehabilitation device similar to the Xbox Kinect, using the same Kinect sensor to track a user's movement in space, but with rehabilitation exercises gamified with customisability.
4. Humac balance system, CSMi Solutions, Stoughton, Massachusetts, USA	A rehabilitation specific device that is similar to the WiiFit by using a balance board to provide feedback about centre of pressure under the feet. The games and exercises are simpler and slower than the WiiFit and enables customisation.
5. LusioMate, Lusio Rehab, Sydney, Australia	A sensor-based wearable gaming control which detects movements of joints and therefore the participant can play games while doing a single joint movement or activity.

6. StepWatch Activity Monitor paired with clinical application on tablet or smartphone, Modus Health, Edmonds, Washington, USA	A small rehabilitation device that is worn around ankle that measures number of steps taken per day. Clinical application allows the user to receive feedback on step count in real time.
7. Applications on iPad and iPhone:	Applications that allow specific exercises to be chosen and scheduled for users according to their specific needs. E.g., PhysioTherapy eXercises (PTX) mobile application, Sydney, Australia; Clock Yourself application, Brisbane, Australia.
