# Supplementary material for: Implementing digital devices to increase mobility training for people receiving inpatient rehabilitation: protocol for a feasibility hybrid type II randomized controlled trial
Source: Pilot Feasibility Stud. 2023 Apr 25;9:69. doi: 10.1186/s40814-023-01298-y (PMC10126551; doi:10.1186/s40814-023-01298-y)
Supplement: Supplementary file 4 — Additional file 4.Therapist-participant questionnaire to explore determinants of implementation success. Questionnaire used to capture the physiotherapists change in capability, opportunity, and motivation to prescribing and delivering rehabilitation using digital devices, developed based upon the COM-B self-evaluation questionnaire. [file 40814_2023_1298_MOESM4_ESM.pdf]

ADDITIONAL FILE 4

Therapist-participant questionnaire to explore determinants of implementation success:

Thinking about the time since the first technology workshop please circle yes or no or rate on a scale of 1 to 5 your agreement with the following statements

1. strongly disagree;

2. disagree;

3. neither agree or disagree (neutral);

4. agree;

5. strongly agree.

Please circle your answer

Since the technology workshop

		1 (strongly disagree) to 5 (strongly agree)				
1	I have had sufficient access to a range of technologies to continue to practice using technologies in rehabilitation	1	2	3	4	5
2	I have increased my confidence to use a range of technologies with my patients in rehabilitation	1	2	3	4	5
3	I have utilised the intervention protocol to use the technologies with my patients in rehabilitation		Yes		No	
4	I have utilised the clinical champion to use the technologies with my patients in rehabilitation		Yes		No	

5	I have attended clinical reasoning sessions to discuss case studies using technology	Yes No
6a	I have had technical difficulties with using at least one of the technologies	Yes No- go to question 7
6b	I was able to manage the technical difficulty myself	Yes- go to question 7 No
6c	I had sufficient support to manage the technical difficulty	Yes No
7a	I have used the Nintendo Wii with ≥ 1 patient	Yes No- go to question 8
7b	I am confident using the features of the Nintendo Wii in my practice	1 2 3 4 5
7c	I am confident using the Nintendo Wii for therapeutic benefit	1 2 3 4 5
8a	I have used the Xbox Kinect with ≥ 1 patient	Yes No- go to question 9
8b	I am confident using the features of the Xbox Kinect in my practice	1 2 3 4 5
8c	I am confident using the Xbox Kinect for therapeutic benefit	1 2 3 4 5
9a	I have used the Humac with ≥ 1 patient	Yes No- go to question 10
9b	I am confident using the features of the Humac in my practice	1 2 3 4 5
9c	I am confident using the Humac for therapeutic benefit	1 2 3 4 5
10a	I have used Fysiogaming with ≥ 1 patient	Yes No- go to question 11

10b	I am confident using the features of the Fysiogaming in my practice	1	2	3	4	5
10c	I am confident using the Fysiogaming for therapeutic benefit	1	2	3	4	5
11a	I have used Step watch with ≥ 1 patient	Yes		No- go to question 12		
11b	I am confident using the features of the Step watch in my practice	1	2	3	4	5
11c	I am confident using the Step watch for therapeutic benefit	1	2	3	4	5
12a	I have used PTexercises on iPad with ≥ 1 patient	Yes		No- go to question 13		
12b	I am confident using the features of the PTexercises on iPad in my practice	1	2	3	4	5
12c	I am confident using the PTexercises on iPad for therapeutic benefit	1	2	3	4	5
**Any other comments?**						

This survey is based on the COM-B self-evaluation Questionnaire; Michie S, Atkins L, West R. The Behaviour Change Wheel: a guide to designing interventions. Great Britain: Silverback Publishing; 2014.
